# Supplementary material for: The role of learned song in the evolution and speciation of Eastern and Spotted towhees
Source: PLoS Comput Biol. 2025 Jun 17;21(6):e1013135. doi: 10.1371/journal.pcbi.1013135 (PMC12204620; doi:10.1371/journal.pcbi.1013135)
Supplement: S1 Text — Table A. Additional information about “hybrid/unsure” recordings. Information provided in Macaulay Library and eBird by recordists to accompany recordings in which the observer suspected a hybrid Eastern × Spotted towhee or was unsure whether the bird was an Eastern or Spotted towhee. Table B. Associations between song feature data and recording metadata. Results for generalized linear models of song feature data against latitude, longitude, species classification, and conversion status (determined by whether the audio file type and/or sampling rate was changed from the original song file). These analyses include only recordings from breeding season months. P-values that are bolded and highlighted in green are below an α = 0.05 threshold. Table C. Results for machine learning models for analyses of song feature data from the subset of recordings of Spotted and Eastern towhees that were obtained during the breeding season. P-values that are bolded and highlighted in green are below α = 0.05. Table D. Prediction accuracies of random forest models trained on 16 song features from samples of Spotted towhees and Eastern towhees using different numbers of decision trees. (A-C) Predictions of subset of all song samples (Ntest = 697) trained on song data from the entire geographic range (NSpotted_towhee = 796; NEastern_towhee = 796). (D-F) Models trained on a subset of samples obtained from the non-overlap zone (NSpotted_towhee = 796; NEastern_towhee = 796). The model was tested on a random subsample of song bouts from both the zone of non-overlap (Ntest_nonoverlap = 216) and the zone of overlap (Ntest_overlap = 216). Increasing the number of trees did not substantially change the accuracy of the models’ predictions. We report the results from models with 500 trees in the main text. Fig A. Frequency of song recordings of each species by longitude. Line plot of the fraction of Spotted towhee and Eastern towhee song recordings, calculated as the number of recordings of each s [file pcbi.1013135.s001.pdf]

S1 Text for "The role of learned song in the evolution and speciation of Eastern and Spotted towhees"

ML audio Recording ID of putative hybrid (H) or unsure (U)	Date	Latitude	Longitude	Number of observers	Photo(s)	Description from recordist(s)
225556 (H)	23 May 2017	40.6875	-99.3834	3	none	White patch at base of primaries and white spots along scapulars and back.
225569 (H)	29 May 2017	40.2853	-103.6973	3	none	Back spots and primary patch
225572 (H)	29 May 2017	40.3051	-103.6224	3	none	Adult male adult female pair. with extensive spotting on back and prominent white patch at base of primaries. Recorded. The bird's mate can be heard in the background. She was also collected, but the recordings were not good enough to get their own record.
225576 (H)	31 May 2017	40.4146	-103.3787	1	none	Adult male, recorded. Limited spotting on back and scapulars and small white primary patch
62893271 (H)	1 Jul 2017	43.4995	-96.5934	1	none	Sounded like a spotted towhee, but its appearance didn't convince me it was fully an SPTO. Also saw a previous report of a hybrid in recent days
67598691 (U)	22 May 2017	41.4069	-97.0830	3	none	none
67677611 (H)	26 May 2017	41.1022	-101.1073	3	none	White at primary base and extensive spotting on wings and scapulars. Adult male recorded
67686671 (H)	28 May 2017	41.1523	-101.0787	3	none	Spotting on back with white primary patch.
88388061 (H)	16 Jun 2017	46.8657	-96.4676	1	[1]	none
104408311 (H)	14 Jun 2018	46.8657	-96.4676	1	[2]	Appeared to look like a Spotted Towhee, however this individual sang the classic "drink your tea" song of an Eastern. Photos and audio taken.
104692741 (H)	16 Jun 2018	40.8378	-102.8065	2	none	ID of calling towhee never ascertained.<br><br>3 females and 2 males. 2 females close to EATO but with a couple scattered white spots on scaps. Others intermediate spotting between montanus SPTO and Eastern Towhee. Recorded singing
115241591 (U)	5 Jun 2018	40.2853	-103.6973	4	none	Birds seen were mostly P. maculatus, but had a small amount of white at bases of central primaries.
154234301 (H)	24 Apr 2019	38.8439	-97.5868	1	[3]	Continuing individual. This time seen up by the discovery center. Photos and audio to come.
165892761 (H)	25 Jun 2019	40.8378	-102.8065	2	[4]	Audio of the bird in photos. Apparently I can open my program for editing files or I'd edit out my talking at the beginning. I'll try to remedy that.
236017491 (H)	14 May 2020	42.4086	-71.4724	1	none	none
242433591 (H)	10 Jun 2020	43.0606	-96.4668	1	[5]	Heard its call and found the bird. Photos and audio.
284225301 (H)	30 Nov 2020	42.6411	-70.8202	1	[6]	Male; pics & audio. Seen and heard (singing!) from parking lot in corner of parking lot, approximately here: 42.6412496, -70.8208768. Bird's vocalizations drew my attention as I returned to lot from trails (note: all vocalizations were unsolicited). I found the bird perched in a thicket at about head height, where it continued to vocalize a bit; it only stayed a few minutes, then it went out of sight (I think around the corner). Song seems to me to be a version of Spotted. Abutter said bird has been at private, inaccessible feeder (which is not visible from road) for couple of weeks. Abutter is very nice but seems to desire privacy; please respect. Thanks to Sean Williams, Marshall Iliff, and Jan Smith for their help with the ID! Some notes from them: The white spots on the back and coverts (though seems a bit muted) are good for Spotted, while the white bases to the primaries (though muted) are good for Eastern. So it's a pretty classic-looking hybrid. The white wing check needs to be absent for it to be a pure Spotted since that trait is only found in Eastern and tends to be dominant such that it appears on all f1 hybrids.
333371791 (U)	3 May 2021	38.2446	-107.7607	none	none	Eastern Towhee like song. Adjacent Spotted Towhees were singing typical songs. Heavy river noise in background.<br><br>Probable Eastern Towhee heard singing "drink your tea" song. I was unable to see the Towhee due to thick brush.
339273711 (H)	14 May 2021	40.8378	-102.8065	1	[7]	Photo and song. Song is most like Spotted. Almost no spotting on back
456940621 (H)	2 Jun 2022	40.4290	-101.5841	1	[8]	Photos and recorded. About 25% spotting typical of SPTO in this area but with long tail spots c/w both EATO and "Arctic" SPTO but not the less-spotted forms from CO mountains (and onto plains)
461508881 (U)	10 Jun 2022	41.9490	-97.6309	3	[9]	observed pair as female with SPTO markings (1st photo below) perched close by and launched into non-stop SPTO "wheeze call" per Pieplow's description (1st audio below) when we neared a large tree next to path with surrounding brush, while male with wing pattern more like EATO (2nd photo below) stayed in the background sneakily carrying food and sparingly singing a musical song more evocative of EATO (also 1st audio below) male #2 was within earshot of the pair and was singing a matching song, but 2 other singers a distance away had differing songs (2nd audio below of male #3 singing a more SPTO-esque song and male #4 heard singing an EATO/SPTO fusion in background of NOPA recordings)
466803501 (U)	6 Jul 2022	40.9849	-98.1501	1	none	none
567583611 (H)	5 May 2023	39.1086	-97.5701	7	none	Call and song resembled spotted, some spots on back with white spot on wing.
592239321 (H)	24 Jun 2023	42.6051	-96.7010	1	none	Looked exactly as a pure Eastern should, and its first songtype was classic Eastern as well. But then its call note was classic Spotted. I have audio recordings.
596898561 (H)	13 Jul 2023	50.5349	-102.3351	3	[10]	I had returned to the hill to find the bird i had heard but couldn't find the day before. When i entered the observation on iNaturalist, 2 identifiers, Peter Taylor and Christian Artuso, explained there are populations of the Spotted x Eastern Towhee in MB that were noted in the MB BBA. https://www.birdatlas.mb.ca/accounts/appendix_en.jsp#TOWH The iNaturalist observation is here: https://inaturalist.ca/observations/174529333
597334041 (H)	25 Jul 2023	40.8378	-102.8065	1	none	Common here, spotted back but singing both songs.
601329651 (H)	7 Aug 2023	38.6126	-104.6771	1	[11]	Photographs; recording. Limited spotting on back and wing coverts; small white patch at base of primaries.

Table A. Additional information about "hybrid/unsure" recordings. Information provided in Macaulay Library and eBird by recordists to accompany recordings in which the observer suspected a hybrid Eastern × Spotted towhee or was unsure whether the bird was an Eastern or Spotted towhee. Links to photos can be found in the reference list.

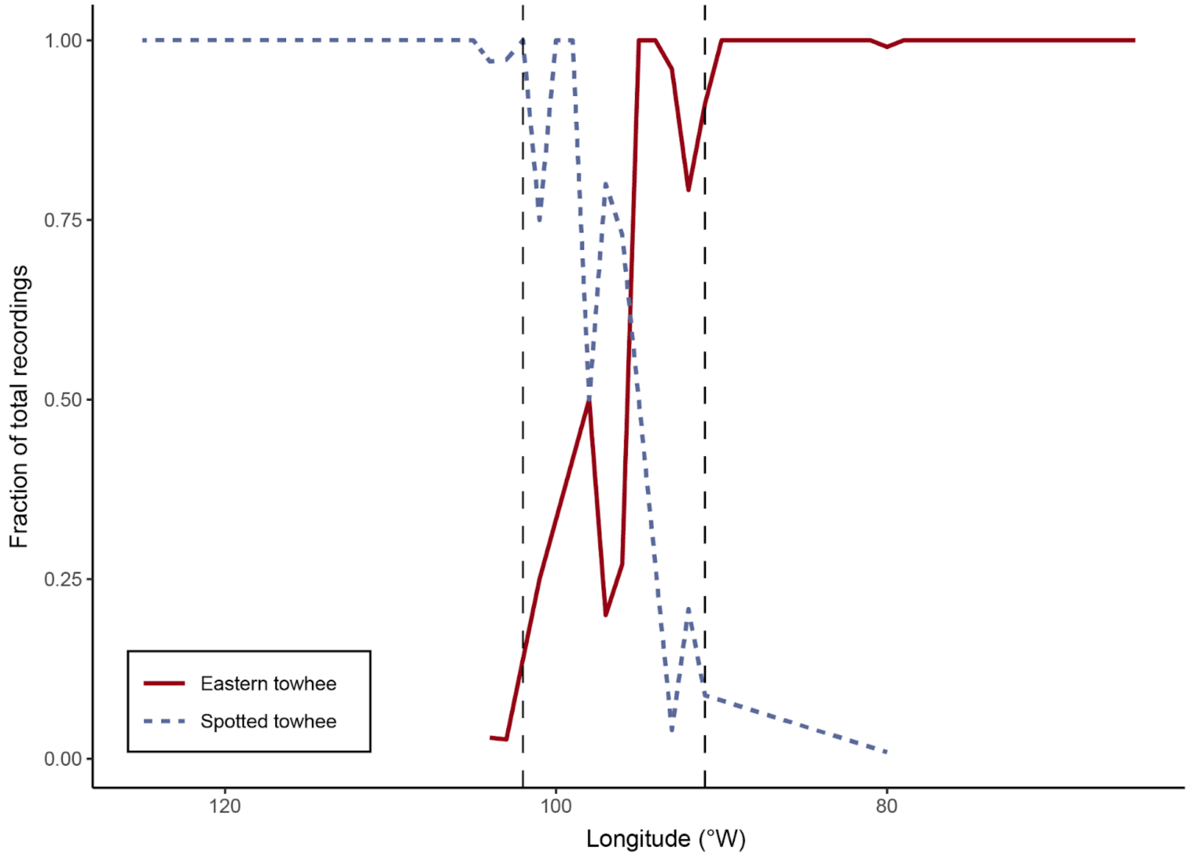

Figure A. Frequency of song recordings of each species by longitude. Line plot of the fraction of Spotted towhee and Eastern towhee song recordings, calculated as the number of recordings of each species divided by the total number of recordings of either species across North America during the breeding season (N_{total}=2785; $N_{Spotted\_towhee}$=1067; $N_{Eastern\_towhee}$=1718). The black dashed vertical lines represent the zone of song overlap, determined based on the co-occurence of song recordings (102°W – 91°W).

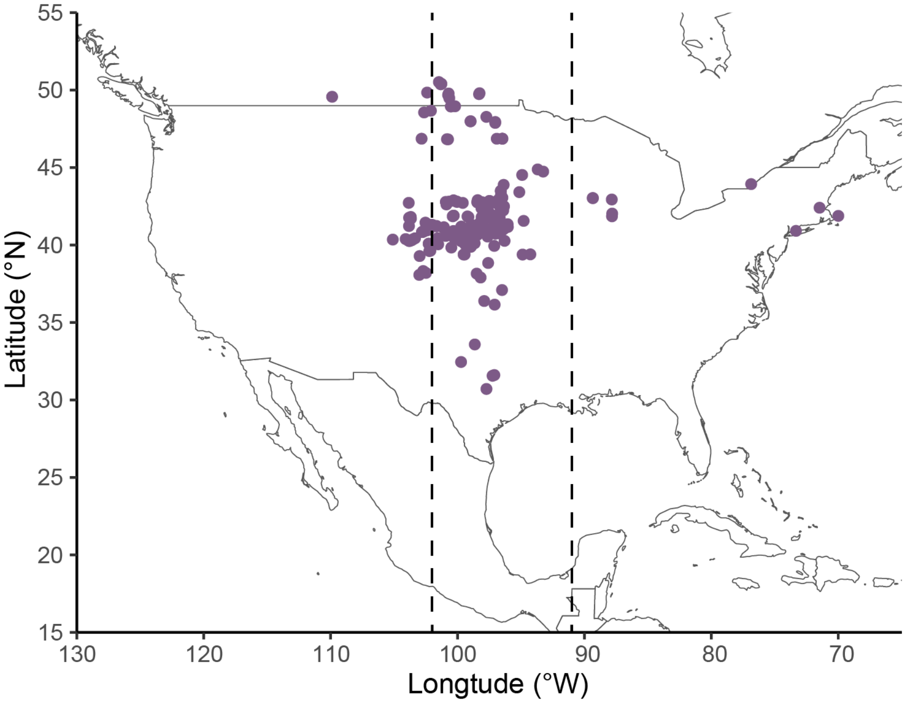

Figure B. Map of putative Spotted towhee × Eastern towhee hybrid sightings (N=203) during the breeding season. 190 of the 203 hybrid sightings in the eBird database had either more than one observer, available media, or observer comments. The dotted line represents the zone of overlap determined by the co-occurence of song recordings (102°W – 91°W); 77.8% of these putative hybrid sightings fall within this zone of song overlap. The sightings of putative hybrids are shifted west compared to the zone of song overlap, potentially suggesting that hybridization is more likely when Eastern towhees are the rarer species. Sighting data obtained from eBird; metadata available at https://github.com/CreanzaLab/TowheeAnalysis. The base map was made with Natural Earth (http://www.naturalearthdata.com/about/terms-of-use/) using the R package naturalearth.

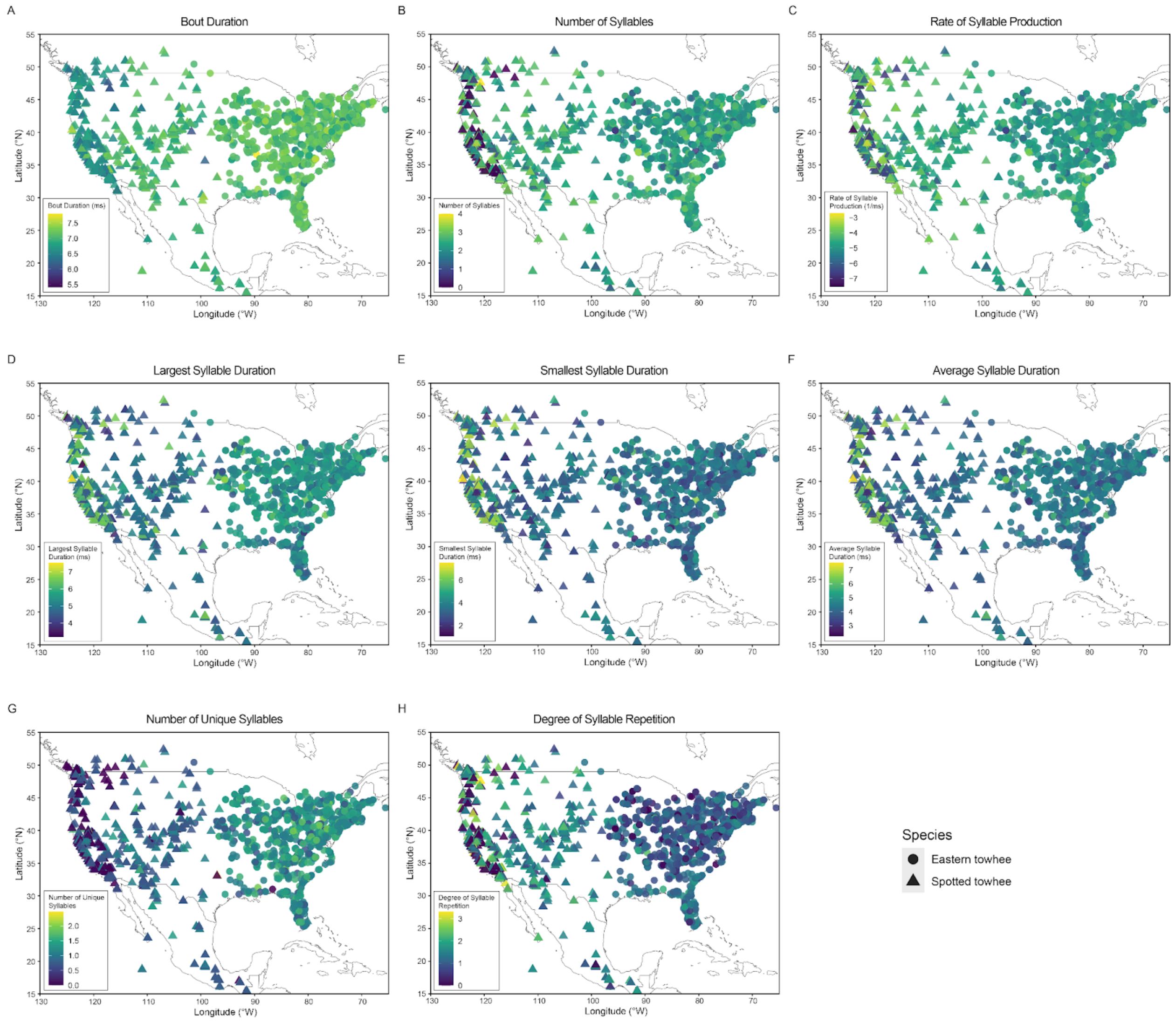

Figure C. Maps of song features related to duration and number of syllables, plotted by their recording location. Each point represents an analyzed song bout (N_{total}=2788; $N_{Spotted\_towhee}$=1069; $N_{Eastern\_towhee}$=1719). The color scale on the bottom left of the map corresponds to the log-transformed song feature value of each recording. The shape of each point indicates the species classification of the recording. Base maps were made with Natural Earth (http://www.naturalearthdata.com/).

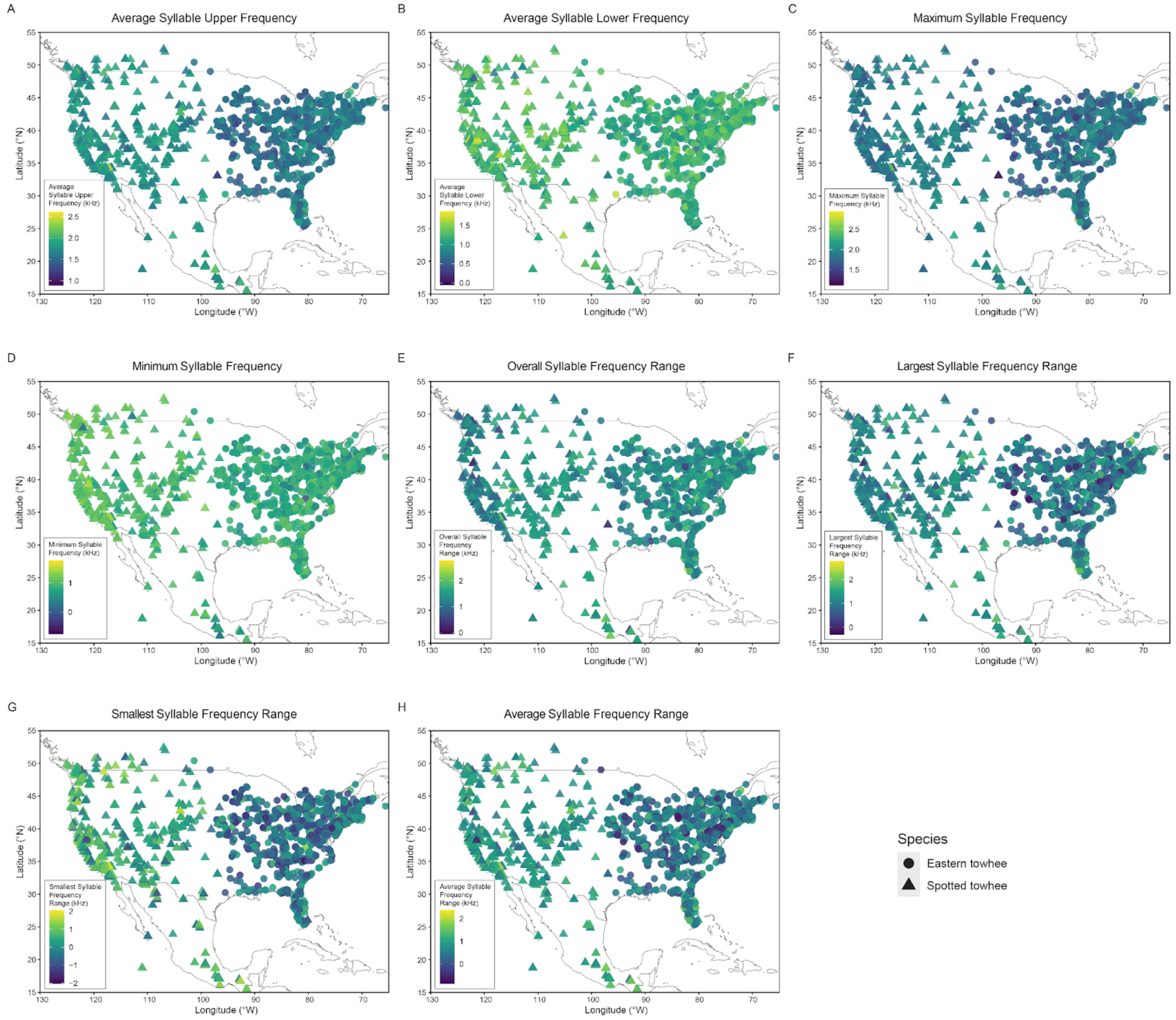

Figure D. Maps of song features related to frequency, plotted by their recording location. Each point represents an analyzed song bout (N_{total}=2788; $N_{Spotted\_towhee}$=1069; $N_{Eastern\_towhee}$=1719). The color scale on the bottom left of the map corresponds to the log-transformed song feature value of each recording. The shape of each point indicates the species classification of the recording. Base maps were made with Natural Earth (http://www.naturalearthdata.com/).

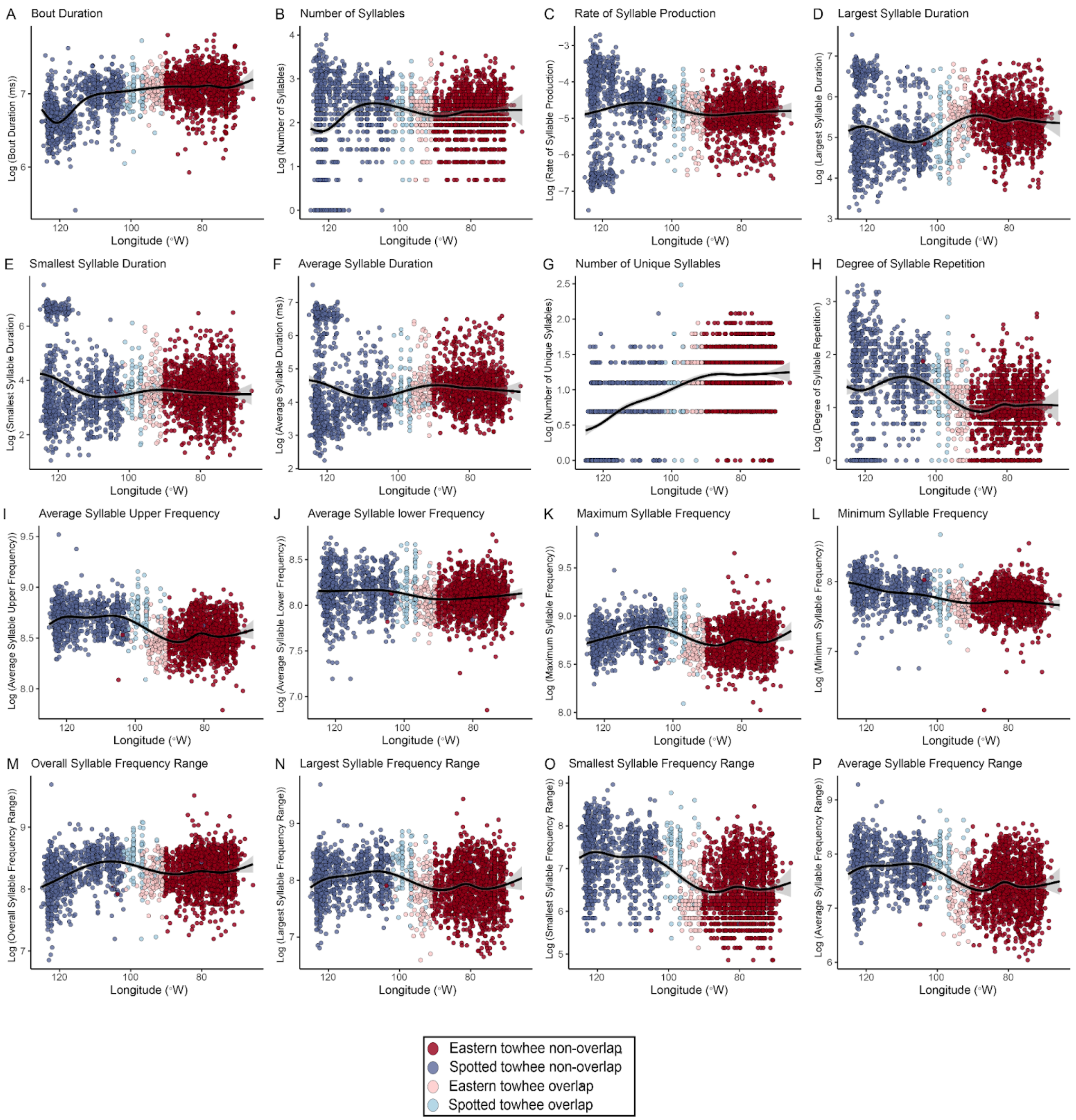

Figure E. Log-transformed song features plotted based on the longitude of the recording location. Each point represents an analyzed song bout (N_{total}=2788; $N_{Spotted\_towhee}$=1069; $N_{Eastern\_towhee}$=1719), with Eastern towhee songs shown in shades of red and Spotted towhee songs in shades of blue. The lighter colors represent recordings from the zone of species overlap. The black line represents a smoothed average using a generalized additive model-fitting method.

Song Feature	Intercept t-value	Intercept p-value	Latitude t-value	Latitude p-value	Longitude t-value	Longitude p-value	Species t-value	Species p-value	Conversion t-value	Conversion p-value
**Bout duration (ms)**	757.96	**$p < 10^{-50}$**	-4.17	**3.15×10^{-5}**	11.06	**8.67×10^{-28}**	-1.25	0.21	-2.81	**5.04×10^{-3}**
**Number of syllables**	91.92	**$p < 10^{-50}$**	-0.77	0.44	2.56	0.01	5.00	**6.06×10^{-7}**	-0.06	0.96
**Rate of syllable production (1/ms)**	-163.20	**$p < 10^{-50}$**	-0.06	0.96	-5.03	**5.23×10^{-7}**	2.23	**0.03**	0.46	0.65
**Largest syllable duration (ms)**	201.78	**$p < 10^{-50}$**	1.40	0.16	-8.57	**1.81×10^{-17}**	-8.65	**9.12×10^{-18}**	0.76	0.45
**Smallest syllable duration (ms)**	44.36	**$p < 10^{-50}$**	-3.35	**8.27×10^{-4}**	-11.92	**7.14×10^{-32}**	-6.32	**3.08×10^{-10}**	1.79	0.07
**Average syllable duration (ms)**	93.96	**$p < 10^{-50}$**	0.10	0.92	-11.06	**8.62×10^{-28}**	-6.43	**1.54×10^{-10}**	1.41	0.16
**Number of unique syllables**	79.09	**$p < 10^{-50}$**	-0.88	0.38	5.86	**5.22×10^{-9}**	-7.00	**3.23×10^{-12}**	-2.06	**0.04**
**Degree of syllable repetition**	35.81	**$p < 10^{-50}$**	1.54	0.12	-3.32	**9.06×10^{-4}**	5.85	**5.49×10^{-9}**	-1.24	0.22
**Average syllable upper frequency (Hz)**	1279.00	**$p < 10^{-50}$**	-4.45	**9.16×10^{-6}**	3.78	**1.59×10^{-4}**	15.10	**2.48×10^{-49}**	0.43	0.67
**Average syllable lower frequency (Hz)**	975.46	**$p < 10^{-50}$**	1.48	0.14	2.69	**7.26×10^{-3}**	6.94	**4.94×10^{-12}**	-3.27	**1.08×10^{-3}**
**Maximum syllable frequency (Hz)**	1192.65	**$p < 10^{-50}$**	-5.75	**9.86×10^{-9}**	9.15	**1.13×10^{-19}**	12.64	**1.68×10^{-35}**	-1.63	0.10
**Minimum syllable frequency (Hz)**	940.84	**$p < 10^{-50}$**	3.51	**4.60×10^{-4}**	-7.44	**1.40×10^{-13}**	2.40	**0.02**	-2.72	6.54E-03
**Overall syllable frequency range (Hz)**	657.63	**$p < 10^{-50}$**	-6.91	**6.25×10^{-12}**	11.44	**1.44×10^{-29}**	10.88	**6.15×10^{-27}**	-0.56	0.57
**Largest syllable frequency range (Hz)**	476.01	**$p < 10^{-50}$**	-5.70	**1.31×10^{-8}**	4.47	**8.07×10^{-6}**	8.23	**2.92×10^{-16}**	2.11	**0.03**
**Smallest syllable frequency range (Hz)**	212.38	**$p < 10^{-50}$**	-3.41	**6.60×10^{-4}**	-4.45	**8.83×10^{-6}**	7.36	**2.52×10^{-13}**	3.45	**5.64×10^{-4}**
**Average syllable frequency range (Hz)**	395.46	**$p < 10^{-50}$**	-5.26	**1.57×10^{-7}**	1.44	0.15	8.94	**7.38×10^{-19}**	2.96	**3.15×10^{-3}**

Table B. Associations between song feature data and recording metadata. Results for generalized linear models of song feature data against latitude, longitude, species classification, and conversion status (determined by whether the audio file type and/or sampling rate was changed from the original song file). These analyses include only recordings from breeding season months. P-values that are bolded and highlighted in green are below an $\alpha=0.05$ threshold.

Breeding Season Analysis	Description	Overall Accuracy	Balanced Accuracy	Cohen's kappa (κ)	Permutation Test P-Value (*p*)
Deep Learning	N_{train}=1304 (652 eastern; 652 spotted)<br>N_{test}=609<br><br>Raw song feature data was centered and scaled.	0.908	0.917	0.808	**$p<10^{-3}$**
Gradient Boosting Machine	N_{train}=1304 (652 eastern; 652 spotted)<br>N_{test}=609<br><br>Raw song feature data was centered and scaled.	0.924	0.925	0.839	**$p<10^{-3}$**
Random Forest Model 1	N_{train}=1304 (652 eastern; 652 spotted)<br>N_{test}=609<br><br>Song feature data was log transformed.	0.924	0.928	0.840	**$p<10^{-3}$**
Random Forest Model 2	N_{train}=1304 (652 eastern; 652 spotted)<br>$N_{test\_nonoverlap}$=167<br>$N_{test\_overlap}$=167<br><br>Song feature data was log transformed. Model was trained on a subset of bouts from the non-overlap region and tested on a subset of non-overlap bouts and all the samples from the overlap region.	overlap=0.874<br><br>non-overlap =0.928	overlap=0.850<br><br>non-overlap =0.921	overlap=0.722<br><br>non-overlap =0.850	**overlap=$p<10^{-3}$**<br><br>**non-overlap =$p<10^{-3}$**
Linear Discriminant Analysis	N_{train}=1304 (652 eastern; 652 spotted)<br>N_{test}=609<br><br>Used raw song feature data.	0.906	0.909	0.802	**$p<10^{-3}$**
Linear Discriminant Analysis on Principal Components 1 & 2	N=2436 (1562 eastern; 874 spotted)<br><br>Song feature data was log transformed. Data is also centered and scaled.	0.761	0.727	0.466	**$p<10^{-3}$**
Uniform Manifold Approximation and Projection + Linear Discriminant Analysis	N=2436 (1562 eastern; 874 spotted)<br><br>Song feature data was log transformed. Data is also centered and scaled.<br><br>(results for each version of the UMAP of different "min_dist" and "n_neighbors" is in the R Script)	0.849	0.841	0.676	**$p<10^{-3}$**

Table C. Results for machine learning models for analyses of song feature data from the subset of recordings of Spotted and Eastern towhees that were obtained during the breeding season. P-values that are bolded and highlighted in green are below α=0.05.

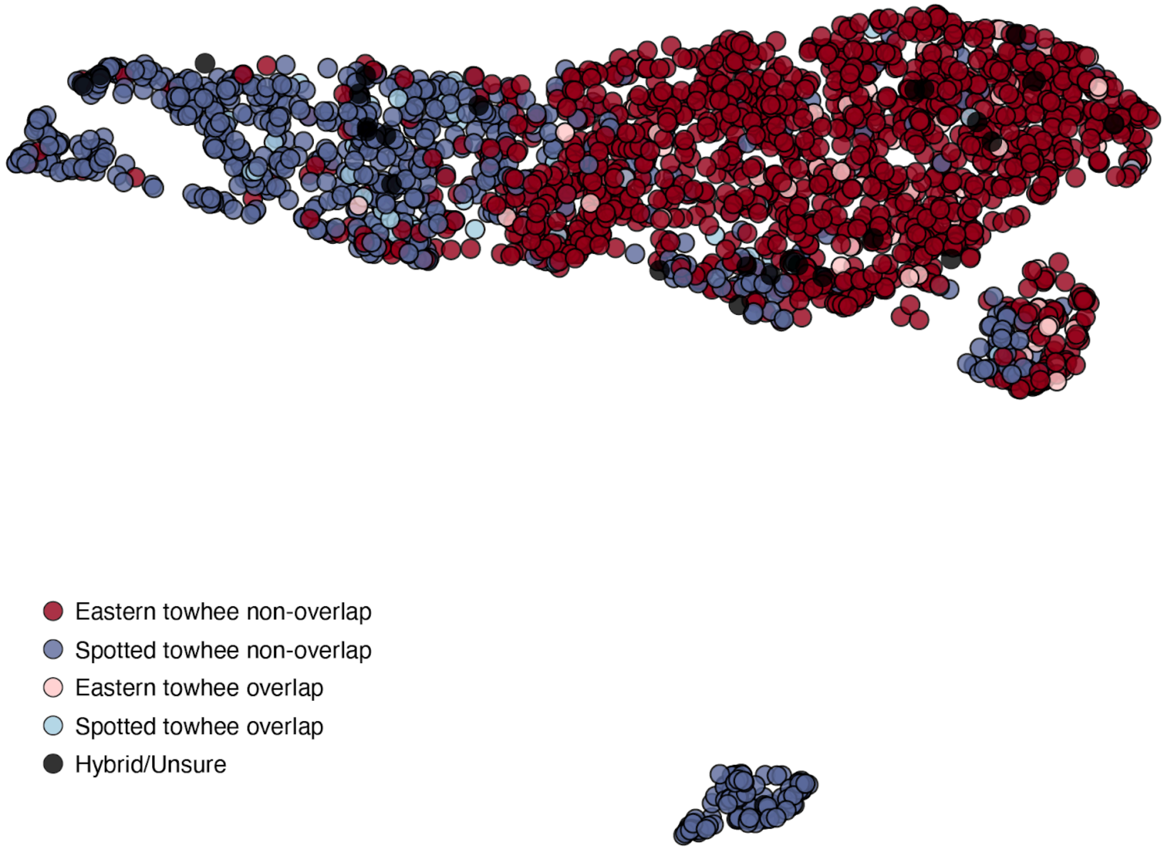

Figure F. UMAP projection of Eastern and Spotted towhee song-feature data using the subset of samples recorded during the breeding season. Each point represents an analyzed song bout (N_{total_bouts}=2436; $N_{Spotted\_towhee}$=874; $N_{Eastern\_towhee}$=1562), with Eastern towhee songs shown in shades of red and Spotted towhee songs in shades of blue. The lighter colors represent recordings from the zone of species overlap. Black dots indicate the 26 recordings from individuals that were classified as potential hybrids ("hybrid/unsure"). Using a linear discriminant classifier to partition the UMAP projection into two sections, we could accurately predict the species of 84.9% of recordings (n_neighbors=15 and min_dist=0.1). Using values of n_neighbors up to 50 and values of min_dist up to 0.9, the linear classifier showed prediction accuracies ranging from 81.3% to 86.4%.

Model	Training Data	Number of Trees	Accuracy	Balanced Accuracy
A	All bout samples	100	89.4%	90.0%
B	All bout samples	500	89.5%	90.1%
C	All bout samples	1000	89.1%	89.7%
D	Zone of non-overlap	100	92.6% (non-overlap)<br>84.3% (overlap)	92.7% (non-overlap)<br>83.4% (overlap)
E	Zone of non-overlap	500	93.1% (non-overlap)<br>84.3% (overlap)	95.8% (non-overlap)<br>83.4% (overlap)
F	Zone of non-overlap	1000	93.5% (non-overlap)<br>84.3% (overlap)	93.6% (non-overlap)<br>83.4% (overlap)

Table D. Prediction accuracies of random forest models trained on 16 song features from samples of Spotted towhees and Eastern towhees using different numbers of decision trees. (A-C) Predictions of subset of all song samples (N_{test}=697) trained on song data from the entire geographic range ($N_{Spotted\_towhee}$=796; $N_{Eastern\_towhee}$=796). (D-F) Models trained on a subset of samples obtained from the non-overlap zone ($N_{Spotted\_towhee}$=796; $N_{Eastern\_towhee}$=796). The model was tested on a random subsample of song bouts from both the zone of non-overlap ($N_{test\_nonoverlap}$=216) and the zone of overlap ($N_{test\_overlap}$=216). Increasing the number of trees did not substantially change the accuracy of the models' predictions. We report the results from models with 500 trees in the main text.

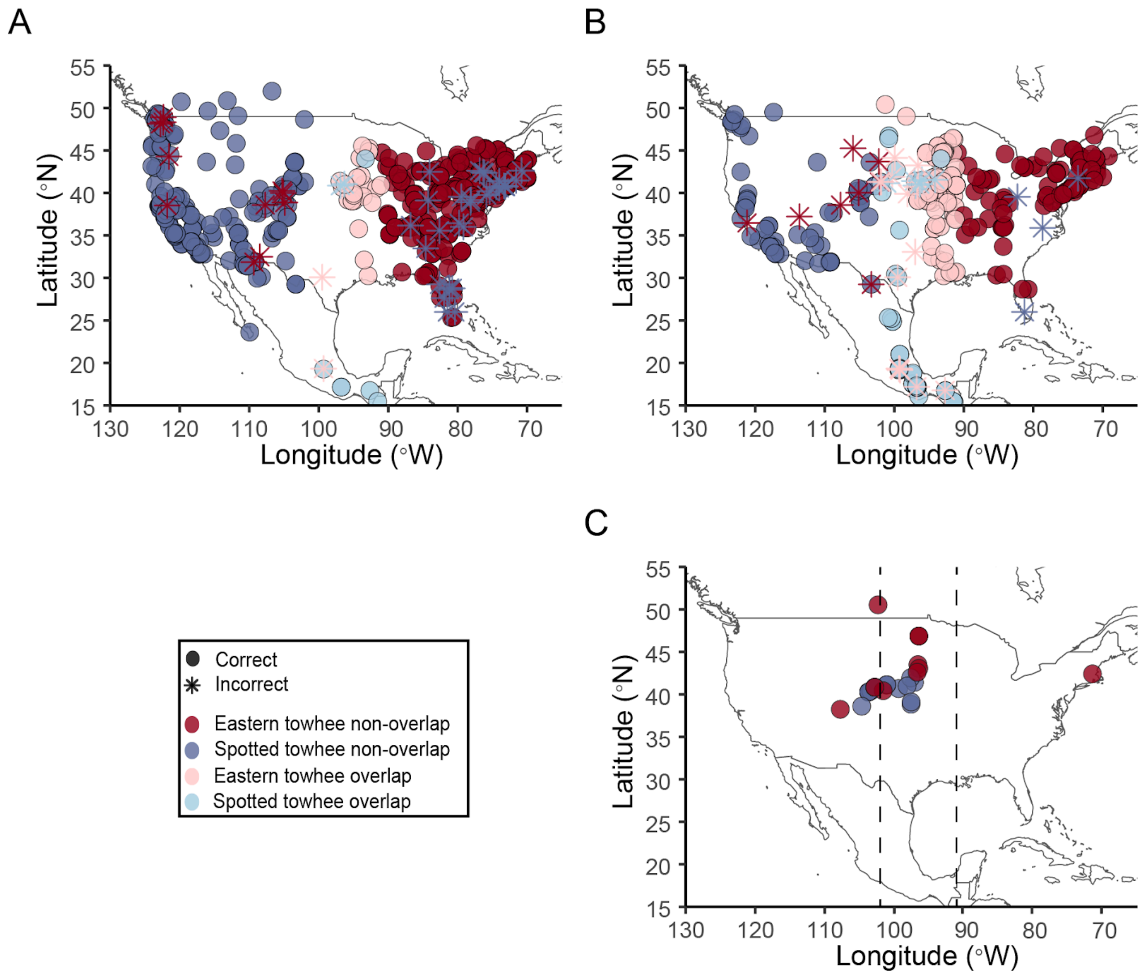

Figure G. Geographic distribution of species predictions using a random forest model trained on 16 song features from only those samples of Spotted towhees and Eastern towhees that were recorded during the breeding season. (A) We trained a model on song data from the entire geographic range of songs recorded during the breeding season ($N_{Spotted\_towhee}$=652; $N_{Eastern\_towhee}$=652) and tested how well it predicted the species identification of a subset of breeding-season song samples (N_{test}=609; accuracy=92.4%). (B) We then trained a second model on a subset of samples obtained during the breeding season from the non-overlap zone ($N_{Spotted\_towhee}$=652; $N_{Eastern\_towhee}$=652). This model was tested on a random subsample of breeding-season song bouts from both the zone of non-overlap ($N_{test\_nonoverlap}$=167; accuracy=92.8%) and the zone of overlap ($N_{test\_overlap}$=167; accuracy=87.4%). (C) We used the same model from panel **B** to predict species identity of song bouts from recordings of "hybrid/unsure" towhees ($N_{predict}$=26). The model predicted that 16 of these "hybrid/unsure" recordings were Spotted towhees and 10 were Eastern towhees, with no discernable longitudinal gradient in the predictions. The dotted line represents the zone of overlap determined by the co-occurence of Eastern

towhee and Spotted towhee song recordings (102°W - 91°W). Base maps were made with Natural Earth (http://www.naturalearthdata.com/).

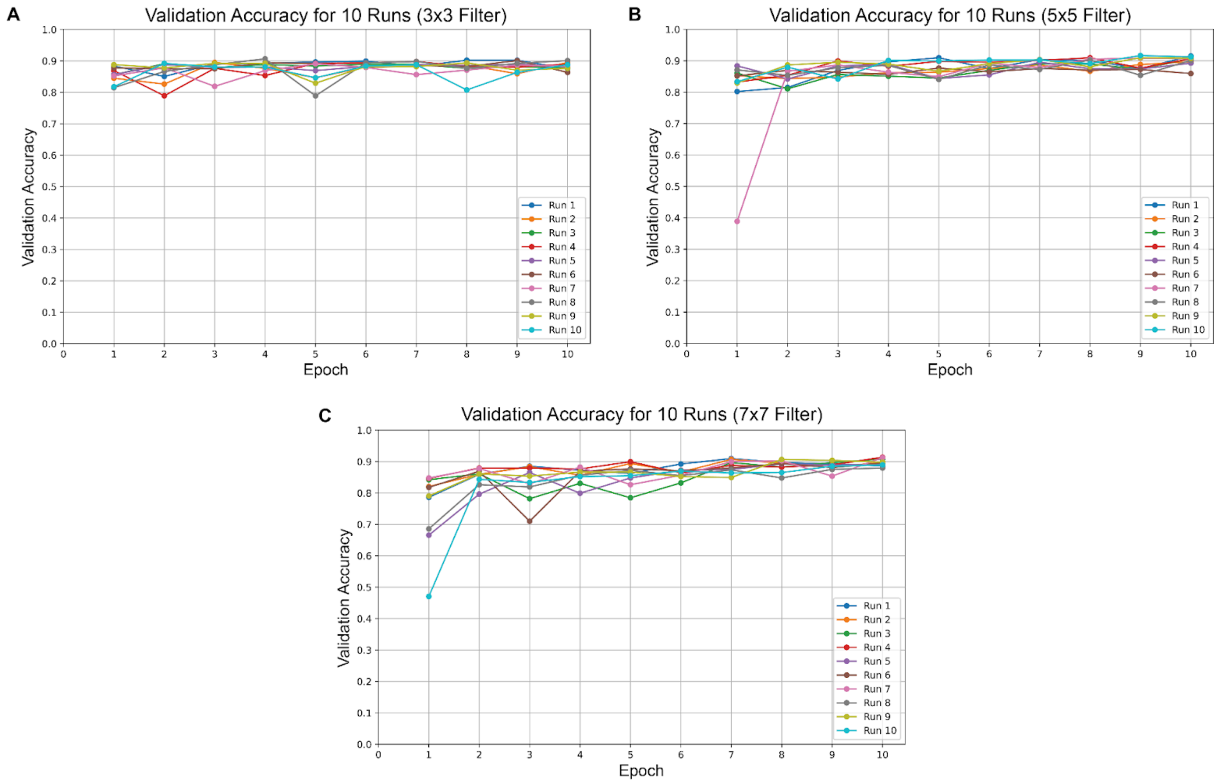

Figure H. Validation accuracies of 10 runs for a convolutional neural network trained on spectrogram images of song recordings of Spotted towhees and Eastern towhees. We trained a model on spectrogram images of song recordings from the entire geographic range of both species ($N_{Spotted\_towhee}$=796; $N_{Eastern\_towhee}$=796) and tested how well it predicted the species identification of a subset of all song samples (N_{test}=697). We passed the entire training dataset through the network 10 times using (**A**) 3x3 pixel filters (average accuracy of last epoch=88.32% ± 1.02%), (**B**) 5x5 pixel filters (average accuracy of last epoch=90.06% ± 1.52%), and (**C**) 7x7 pixel filters (average accuracy of last epoch=89.74 % ± 1.07%).

Supplemental Methods

Because of the potential effects of converting the file type and/or sampling rate of song recordings of the Spotted and Eastern towhee, we modified all of our analyses to incorporate conversion status to assess whether these modifications to the audio files were affecting the predictions of our machine learning models for species classification.

<u>Principal Component Analysis</u>

We conducted a principal component analysis (PCA) in which conversion status was used as a feature alongside the original 16 song features, and we found that conversion status was the second least important feature in PC1 and the least important feature in PC2 . When we fit a linear discriminant analysis (LDA), our species-prediction accuracy remained the same (accuracy=76.2%; balanced accuracy=74.0%; Cohen's κ=0.488; $p < 10^{-3}$). Additionally, when color-coding the PC plot by conversion status, there is significant overlap with no apparent separation between whether recordings were converted or not (**Fig. I**). We also applied a generalized linear model (GLM) on principal components 1 and 2 with conversion status, scaled longitude, scaled latitude, and species as fixed effects and found that conversion status was *not* statistically significantly associated with PC1 (t-value=1.11; p=0.265) or PC2 (t-value=1.10; p=0.271), but longitude and species were significantly associated with both PC1 and PC2 and latitude was significantly associated with PC2.

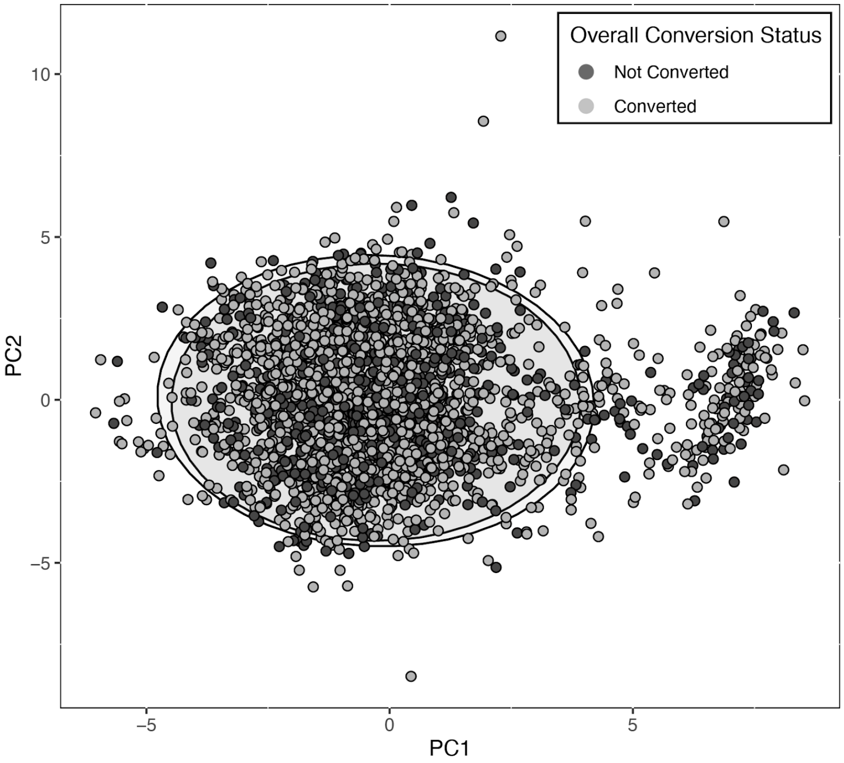

Figure I. Principal component analysis of song bouts using 16 song features. Each point represents an analyzed song bout ($N_{total\_recordings}$=2785; $N_{Spotted\_towhee}$=1067; $N_{Eastern\_towhee}$=1718), color-coded by conversion status. 'Converted' denotes samples in which the original recording file was converted to a .wav file (from .mp3 or .m4a) and/or the sampling rate was converted to 44,100 Hertz. Ellipses indicate 95% confidence intervals.

UMAP

Similarly, we then repeated UMAP analysis, this time including conversion status as a feature in the UMAP. When we used LDA to partition the UMAP space to best distinguish Spotted and Eastern towhees, we found that the accuracy changed only by 0.012 (accuracy=85.2%; balanced accuracy=84.4%; Cohen's κ=0.687; $p < 10^{-3}$). As with the first two principal components as described above, we applied a GLM to the two dimensions of the

UMAP projection and found that conversion status, again, was *not* statistically significant for both UMAP1 (t-value=-0.269; p=0.788) and UMAP2 (t-value=-1.90; p=0.058), but species and longitude were significantly associated with UMAP1 and UMAP2, respectively. When color-coding the UMAP plot by conversion status, there is significant overlap with no apparent separation between whether recordings were converted or not (**Fig. J**).

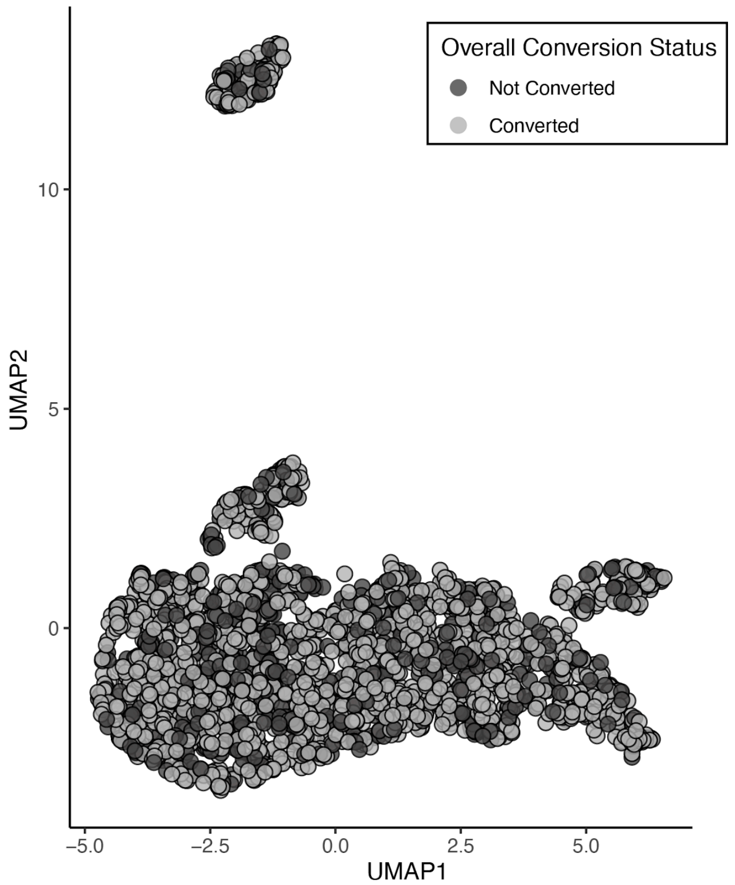

Figure J. UMAP projection of Eastern and Spotted towhee song-feature data.

Each point represents an analyzed song bout (N_{total_bouts}=2785; $N_{Spotted\_towhee}$=1067; $N_{Eastern\_towhee}$=1718; $N_{hybrid/unsure}$=27), color-coded by conversion status. 'Converted' denotes samples in which the original

recording file was converted to a .wav file (from .mp3 or .m4a) or the sampling rate was converted to 44,100 Hertz.

Linear Discriminant Analysis on raw song features

In the LDA using raw song feature data, we incorporated conversion status as an additional feature, and we found that the species-prediction accuracy stayed the same (accuracy=86.8%; balanced accuracy=86.8%; Cohen's κ=0.726; *p*=0.0476). Again, we found that while there was some visible separation of species classification in one-dimensional space, there was still considerable overlap between the 2 species. When color-coding the LD1 plot by conversion status, there is significant overlap with no apparent separation between whether recordings were converted or not (**Fig. K**).

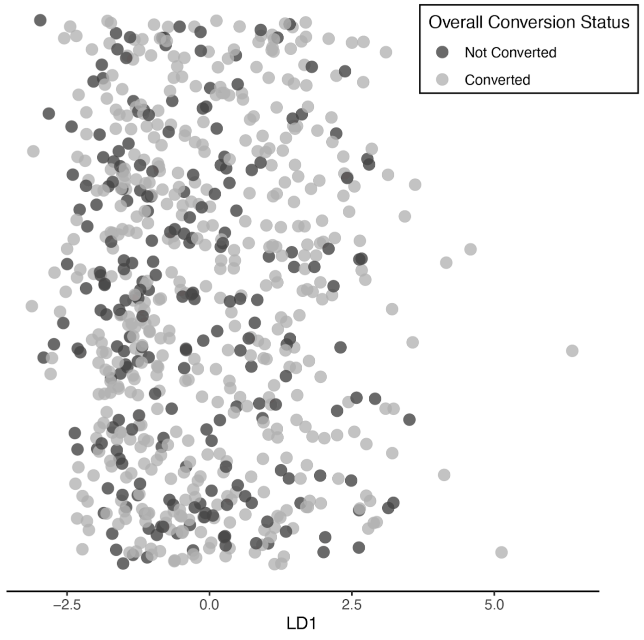

Figure K. Results of a Linear Discriminant Analysis trained on towhee song data. Plot of LD1 results of a subset of towhee song bouts (N_{test}=697) using a Linear Discriminant Analysis trained on raw song

feature data from a balanced training set of Spotted and Eastern towhee bouts (N_{train}=1592). The model revealed 86.8% prediction accuracy (balanced accuracy=86.9%; Cohen's Kappa=0.73). Points are jittered vertically for visualization and color-coded by conversion status. 'Converted' denotes samples in which the original recording file was converted to a .wav file (from .mp3 or .m4a) or the sampling rate was converted to 44,100 Hz.

Gradient Boosting Machine

Additionally, we implemented conversion status as a feature in the gradient boosting machine and found that this feature had the lowest importance, changing the species-prediction accuracy of our model by only 0.001 (accuracy=88.1%; balanced accuracy=88.6%; Cohen's κ=0.755; $p < 10^{-3}$).

Random Forest Model 1

We integrated conversion status as a feature in our random forest model 1. We found that overall conversion status was the feature of lowest importance. This model had a 0.3% decrease in accuracy compared to the model in the main text (accuracy=89.2%; balanced accuracy=89.7%; Cohen's κ=0.778; permutation test $p < 10^{-3}$).

Random Forest Model 2

We then added conversion status as a feature in random forest model 2 as well, and we found that overall conversion status was the feature of lowest importance. When making predictions on the non-overlap zone, the accuracy remained the same (accuracy=93.5%; balanced accuracy=93.6%; Cohen's κ=0.865; permutation test $p < 10^{-3}$). When making predictions on samples from the zone of overlap, the accuracy decreased by 1%

(accuracy=83.3%; balanced accuracy=82.6%; Cohen's κ=0.660; permutation test $p < 10^{-3}$), again suggesting it is not an important feature in making predictions.

Assessing the species distribution of converted files

We first confirmed that our processes of saving mp3 and m4a audio files in wav format and of resampling the files at 44,100 Hz did not affect the frequency or timing of the songs by comparing the spectrograms before and after conversion. Then, we assessed whether the file format, source database, or sampling rate showed species-level differences. We found that Eastern towhees in our database were more likely to be downloaded in wav format from Macaulay library and Spotted towhees were more likely to be downloaded in mp3 format from Xeno-canto. Files from both species were most likely to be recorded at 44,100 or 48,000 Hz.

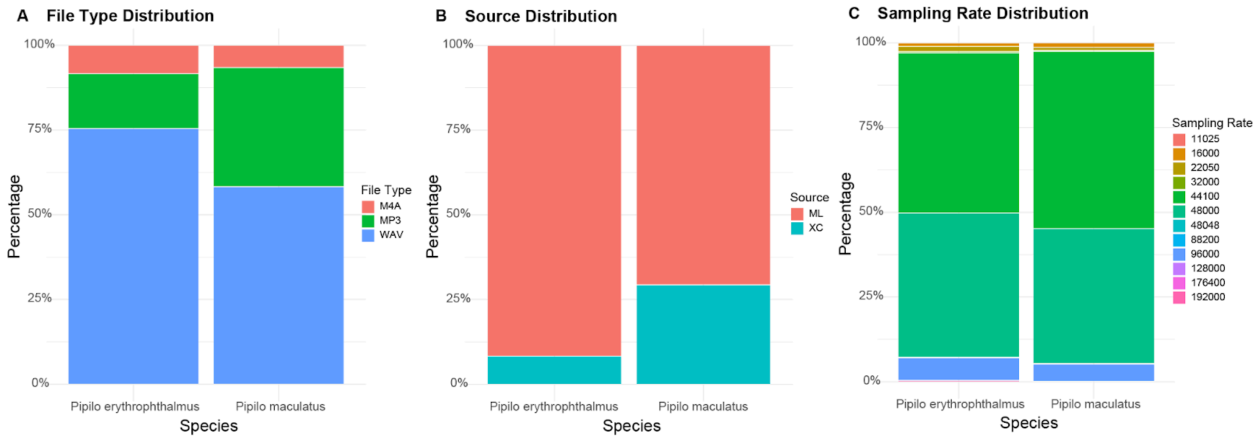

Figure L. Species distributions of different audio file properties. (**A**) We observe that wav files are biased toward the eastern U.S. (and thus the Eastern towhee) and mp3 files are biased toward the western U.S. (and thus the Spotted towhee). (**B**) This file-type bias corresponds to a difference in the proportion of submissions to Macaulay library in the east (which favors wav files) and Xeno-canto in the west (which allows users to download files in mp3 format). (**C**) The vast majority of files were either already sampled at 44,100 Hz (the standard for CD audio) or were sampled at 48,000 Hz (the standard for video).

References

1. https://search.macaulaylibrary.org/catalog?taxonCode=x00446&subId=S43325265&view=list
2. https://search.macaulaylibrary.org/catalog?taxonCode=x00446&subId=S46548196&view=list
3. https://search.macaulaylibrary.org/catalog?taxonCode=x00446&subId=S55374209&view=list
4. https://search.macaulaylibrary.org/catalog?taxonCode=x00446&subId=S57686202&view=list
5. https://search.macaulaylibrary.org/catalog?taxonCode=x00446&subId=S70287998&view=list
6. https://search.macaulaylibrary.org/catalog?taxonCode=x00446&subId=S76902336&view=list
7. https://search.macaulaylibrary.org/catalog?taxonCode=x00446&subId=S88100001&view=list
8. https://search.macaulaylibrary.org/catalog?taxonCode=x00446&subId=S111989428&view=list
9. https://search.macaulaylibrary.org/catalog?taxonCode=y00226&subId=S113489429&view=list
10. https://search.macaulaylibrary.org/catalog?taxonCode=x00446&subId=S145457769&view=list
11. https://search.macaulaylibrary.org/catalog?taxonCode=x00446&subId=S146711504&view=list
