# Supplementary material for: The role of learned song in the evolution and speciation of Eastern and Spotted towhees
Source: PLoS Comput Biol. 2025 Jun 17;21(6):e1013135. doi: 10.1371/journal.pcbi.1013135 (PMC12204620; doi:10.1371/journal.pcbi.1013135)
Supplement: Attachment — Submitted filename: Review_letter.pdf [file pcbi.1013135.s002.pdf]

In this study, authors present an analysis on song variation and divergence between two species of towhee in North America using thousands of citizen-based recordings and three distinct machine learning approaches. They show that there is individual variation in songs and even though there is extensive overlap between species song characters, the classification methods were able to distinguish the two species with a considerable degree of accuracy. Authors concluded that these results may imply that songs could contain sufficient species-specific information to allow behavior discrimination and that songs could have mediated the speciation process.

I think that this is a nice study that will be a valuable contribution to the literature and I commend the authors for a well-done, well-written and well-analyzed manuscript. The weakness of this study is that there is no paired information of songs with morphology and genetics, which would be mostly important at the contact zone between the towhee species where seemingly there is ongoing hybridization. Also, the genetic information is limited both in number of samples, genetic markers and geographic coverage. However, I think the study would be an important contribution and hopefully its publication will trigger more detailed research on genetics and phenotypes at the contact zone. Below I have suggested a few ways that could improve the manuscript further.

General Comments

I like that three different machine learning approaches were used, including the traditional DFA and two new ones. The fact that all three methods generally agree means that results are not idiosyncratic to a specific one. However, I think that the use of three distinct approaches should

be justified in methods. Perhaps because an eventual converge in results would mean that results/patterns are not tied to idiosyncrasies inherent to any individual method?

The authors could estimate the amount of likely admixture in songs in the area of overlap between the two species and across their range (i.e., longitude) as the average posterior probability of song assignments to each towhee species relative to a priory species identity. For example, such posterior probability can be easily obtained in discriminant analyses such as the one performed in the towhee study and plotted against longitude. This would perhaps show that songs with intermediate ancestry are more likely to be found at the contact zone between the two towhee species. See Maldonado-Coelho et al. (2023, citation below) for a similar approach.

Is the number of correctly predicted songs given by each classification method statistically significant? For example, authors could use the statistics Cohen's Kappa to test whether the proportion of correct classifications were significantly higher than expected when individuals had been assigned randomly.

Some of the songs that were correctly predicted to species by classification methods (e.g., random forest) could come from genetically admixed individuals who have learned these songs from one of the parental species at the zone of overlap. This is because oscine passerine hybrids could learn the entire songs from one parental species even though they have and admixed ancestry. They would be genetic hybrids even though their songs are not classified as such. This should be acknowledged or discussed somewhere in the manuscript.

Specific comments

Introduction

Line 44 – replace “birdsong” by “song”

Line 67 – Assortative mating based on songs is a consequence of selection. Rephrase “birds can use their songs for species recognition” since it gives the impression that songs are used with a purpose to avoid hybridization. The same issue is present in line 116: “they are still able to maintain themselves as separate species”.

The authors rely mostly on Price (2007) in the introduction parts that cover the hybrid zone topic. Sure, this is an important reference, but since this is a study on songs that includes a contact zone, at least some of previous works that have assessed song variation at hybrid zones should be included in the framework. Here I suggest a few examples:

Maldonado-Coelho, M., Santos, S. S. D., Isler, M. L., Svensson-Coelho, M., Sotello-Muñoz, M., Miyaki, C. Y., Ricklefs, R. E. and Blake, J. G. 2023. Evolutionary and ecological processes underlying geographic variation in innate bird songs. The American Naturalist 202: E31-E52.

Love, J. and Goller, F. 2021. Processes underlying complex patterns of song trait evolution in a *Setophaga* hybrid zone. Ecol. Evol. 11: 7264–7277.

Kenyon, H. L., M. Alcaide, D. P. L. Toews and D. E. Irwin. 2017. Cultural isolation is greater than genetic isolation across an avian hybrid zone. J. Evol. Biol. 30: 81-95.

Halfwerk, W., C. Dingle, D. M. Brinkhuizen, J. W. Poelstra, J. Komdeur, and H. Slabbekoorn. 2016. Sharp acoustic boundaries across an altitudinal avian hybrid zone despite asymmetric introgression. Journal of Evolutionary Biology 29:1356–1367.

Greig, E. I., and M. S. Webster. 2013. Spatial decoupling of song and plumage generates novel phenotypes between 2 avian subspecies. Behavioral Ecology 24:1004–1013

den Hartog, P. M., S. R. de Kort, and C. ten Cate. 2007. Hybrid vocalizations are effective within, but not outside, an avian hybrid zone. Behavioral Ecology 18:608–614.

Haavie, J., T. Borge, S. Bures, L. S. Garamszegi, H. M. Hampe, J. Moreno,A.Qvarnstrom, J. Torok, andG. P. Sætre. 2004. Flycatcher song in allopatry and sympatry—convergence, divergence and reinforcement. Journal of Evolutionary Biology 17:227–237.

Methods

Line 175 – Define what you mean by syllable segmentation in this section. In line 315 of the results section, you report segmentation as “we segmented the song into syllables”, but this is unclear when reading in the methods. Please provide a short definition in methods.

Line 210 – It is unclear how “overall syllable frequency range” differs from “average syllable frequency range” listed above.

Results

Authors reported that 3515 bouts were used in the analyses. However, from the results section, it becomes clear that in some cases more than one bout was extracted from individual birds. How these repetitions were treated in analyses? Were they averaged for each individual or considered separately? Also, this becomes apparent only when reading the results and this should be explained earlier in methods.

Line 315 – This is part of methods. Importantly, this describes what is meant by segmentation, which is not clear in methods. Please move this sentence there.

Line 322 – How were these birds determined as hybrids? Did authors reviewed photos to confirm their hybrid identity? Or were they only listed as hybrid in public databases? Some objective way to confirm their hybrid identity based on plumage should be provided.

Discussion

Lines 451-452 – It is true that these species are genetically distinct. However, one cannot claim that they are reproductively isolated given either the genetic or acoustic evidence. There are no substantial genetic data from the contact zone and no paired genetic and vocal samples at the individual level from this region to substantiate such statement. Also, the song classification

methods did not recover songs fully to the original species. It is true that songs were correctly assigned to the species with high certainty, but all methods recovered song with less than 90% of certainty. This could imply that there is some level of assortative mating but also there is room for heterospecific mating and therefore reproductive isolation might be incomplete. Also, in line 491, authors conclude that intermediate songs might exist based on the analyses. So, authors need to tone down this claim.

Line 456 – I agree with authors that the song overlap between species recovered in their study, but with specific differences revealed in formal machine learning analyses, may imply that birds themselves were using some cue to discriminate heterospecific from conspecific songs. However, no experiments with playbacks were performed in this study and it would be important to anchor this statement with some literature that have recovered a similar pattern of song overlap and discrimination when using playbacks (e.g. Macedo et al. 2019, Behavioral Ecology 30:1205-1215; https://doi.org/10.1093/beheco/arz066).

Line 476 - The number of unique syllables shows a clinal variation into the range of Spotted tonwhee (Figure 3D). It seems that this could be related to introgression and/or gradients in selective pressures. Authors have not modeled the influence of these factors, but is there any ideas why this pattern exists? In Line 508 authors discuss some of these factors, but I miss a more direct link with specific song aspects.

Line 495 – replace “discourage” by “prevent”

Line 515 – Testing isolation-by-distance could be easily implemented in this study. Also, although it is true that an IBD pattern could be related to gradual changes in cultural drift, such pattern could be explained by gradients of selection (e.g., environment, sexual selection).
