# Supplementary material for: The role of learned song in the evolution and speciation of Eastern and Spotted towhees
Source: PLoS Comput Biol. 2025 Jun 17;21(6):e1013135. doi: 10.1371/journal.pcbi.1013135 (PMC12204620; doi:10.1371/journal.pcbi.1013135)
Supplement: Attachment — Submitted filename: Response to Reviewers (Towhee_LeonCreanza)_updated.pdf [file pcbi.1013135.s004.pdf]

Additional Editor Comments :

Based on my own reading of your revisions, I can see that you have made a lot of efforts in integrating all the previous comments of the three reviewers, particularly with the addition of two new machine learning algorithms. I sent back your revised manuscript to two of the former reviewers, and they have some comments left that would need to be addressed before we can take a decision about your manuscript. Reviewer 3 has only one minor comment left; namely, they would like you to extend the discussion on the effect of the use of mp3 files and necessary conversion. Reviewer 2 raises more comments, notably about the lack of explanations and justifications regarding commands used to run the new algorithms, as well as more details about how you control for the conversion effect you found.

Response: We are thankful to the editor and both reviewers for their thorough reading of our manuscript and the detailed feedback. We have attempted to address all the reviewers' comments and limitations of our study by further clarifying our methodology and discussing them otherwise. We addressed each specific comment below in blue text.

Reviewers' comments:

Reviewer's Responses to Questions

Reviewer #2: General issues
The authors have done a lot of good work to improve the manuscript. Most importantly they do warn about the pitfalls of using a public data source without any real scientific quality control

Response: Thank you for your helpful feedback on our manuscript! We have addressed your comments below.

The text can still be vague in certain locations, please be a bit more specific in your formulations.

Please do not just write what command you used in your software, explain the effect and why you did it. One of many examples is in the Deep Learning section where you state what command you used, not how you used it, what it does, or why you did it. It looks like you implemented cross entropy loss in your classification layer, but that is

normally only done when you have an unbalanced data set. That is not the case with your data.

Response: We have updated the text to include more detailed explanations of our methodology, significantly expanding on the Deep Learning model. From our understanding, when you have a *balanced* data set, cross entropy loss makes more well-calibrated predictions since it is penalizing errors across all classes without favoring the majority class. If you use cross entropy loss on an *unbalanced* dataset, however, it can be a problem since it is more sensitive to the predictions of the majority class which might overwhelm the loss due to the minority class, in which case a weighted cross entropy loss function would likely be more suitable. Since our deep learning model is balanced, we applied the unweighted cross entropy loss to guide our model to learn and improve by adjusting its own predictions based on how well the predicted probabilities align with the true labels.

New Text: "Additionally, we used a deep learning model via the Torch package [45] in R that uses a multilayer perceptron (i.e. fully connected feedforward neural network) for classification of the song bouts. Again, we used a training set consisting of a random balanced subsample of 1592 song bouts ($N_{Spotted\_towhee}$=796 and $N_{Eastern\_towhee}$=796) and tested the model on 697 bouts. The data was centered and scaled prior to integration with the model. The network contains an input layer, two hidden layers, and an output layer. The input layer has a size of 16, defined by the number of song features. We used 64 neurons in the hidden layers, aiming to balance model performance and computational efficiency without overfitting. Each fully connected layer applies a linear transformation to the data, mapping it to a new space using the 'nn_linear' function. The first hidden layer maps the input features to the 64 hidden neurons, followed by a second hidden layer of the same size, both of which use the ReLU activation function to introduce non-linearity. The final fully connected layer outputs the model's raw predictions, which are then passed through a log-softmax activation function to compute class probabilities. The entire training dataset was passed through the model 100 times (num_epochs = 100) during the training process to allow the model to progressively learn from the training data to improve its accuracy and performance. Within each epoch, we used a small batch size of 32 for more stable convergence, such that 32 training samples were used per iteration so that the model frequently updated its parameters during the training process for more efficient training. We used the 'nnf_cross_entropy' loss function for the model to compute the unweighted cross entropy loss for the classification tasks and used an Adam optimization algorithm for Adaptive Moment Estimation with a 0.001 learning rate using the 'optim_adam' function to adjust the model's feature weights during training." L384

Please when you write your response make sure the line numbers are the real line numbers. It makes it hard for us reviewers to find the relevant text when the numbers are wrong.

Response: Thank you for letting us know! In our response, we have made sure to accurately reference the line numbers as it appears on our documents. We are unsure why the PDF looked different on your end. We apologize for this and hope that we were able to accurately reference the line numbers in this response document.

In first round I was concerned about the conversion of sound from MP3 to Wav. Now you have shown it does play a role in your analysis and statistically significantly so. Can you then conclude anything from your tests when you cannot exclude that abiotic factor? As an example, have you investigated how much conversion means for PC1 and PC2? It does not seem like you have tried to isolate the impact of this factor on the various tests, only determined there is a significant effect.

Response: Thank you for this suggestion! We have tried to address your concern in two ways: by further investigating the associations of converted files with other metadata, and by modifying all of our analyses to incorporate conversion status.

First, we would like to illustrate that conversion status appears to play a role in our analyses not because converting the file format or sampling rate of the recordings changes them in ways that cause them to have systematically different song features, but because the files that were converted appear to be geographically biased (and thus biased by species). Below, when we visualize an original file (here, an mp3 with a sampling rate of 22050 Hz), the same file converted to wav format at 22050 Hz, and that wav file resampled at 44100 Hz, we hope it is visually apparent that the frequency and timing of the song remains the same. The spectrogram of the song at 44100 Hz appears crisper because of the increased number of samples that go into the Fourier transform to make the spectrogram, but the songs sound and look the same.

MP3 format, 22050Hz sampling rate

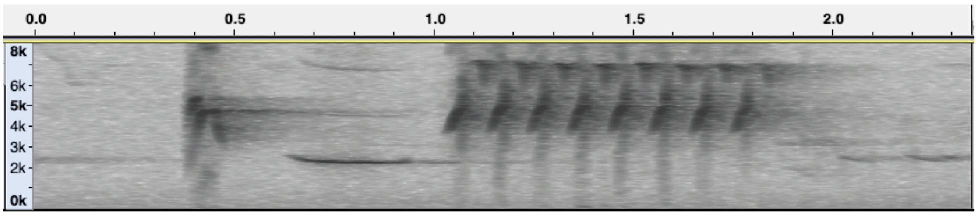

WAV format, 22050Hz sampling rate

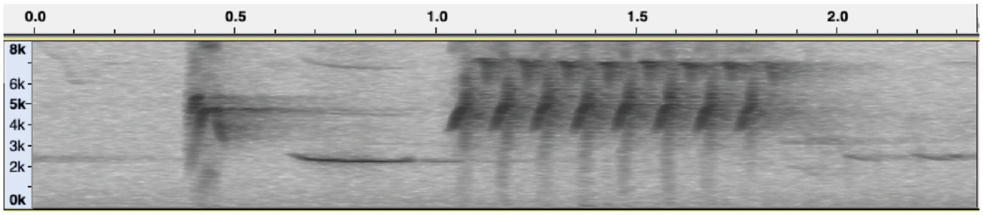

WAV format, 44100Hz sampling rate

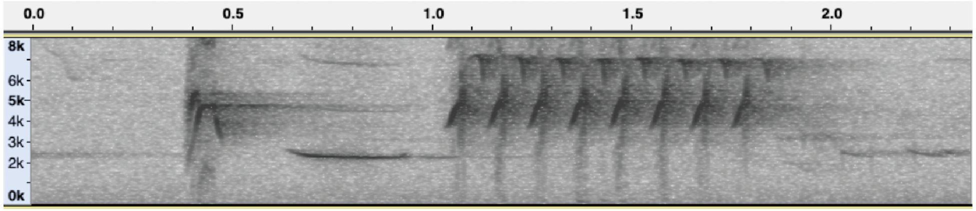

However, when we examine the distribution of songs that needed to be converted, we can see in panel A below that wav files are biased toward the eastern U.S. (and thus the Eastern towhee) and mp3 files are biased toward the western U.S. (and thus the Spotted towhee). This corresponds to a difference in the proportion of submissions to Macaulay library in the east (which favors wav files) and Xeno-canto in the west (which allows users to download files in mp3 format), shown in panel B. The vast majority of files were either already sampled at 44100 Hz (the standard for CD audio) or were sampled at 48000 Hz (the standard for video), panel C. Since any individual song does not have different song features after we converted it, and since the song features we studied show significant geographic variation and/or differences between Eastern and Spotted towhees, the significant results we obtained for five song features when we include conversion status in our generalized linear model appears to be related to the fact that the converted files are not a random sample of files but are instead biased toward the Spotted towhees in the west. This figure is also now in the supplement (Figure S12).

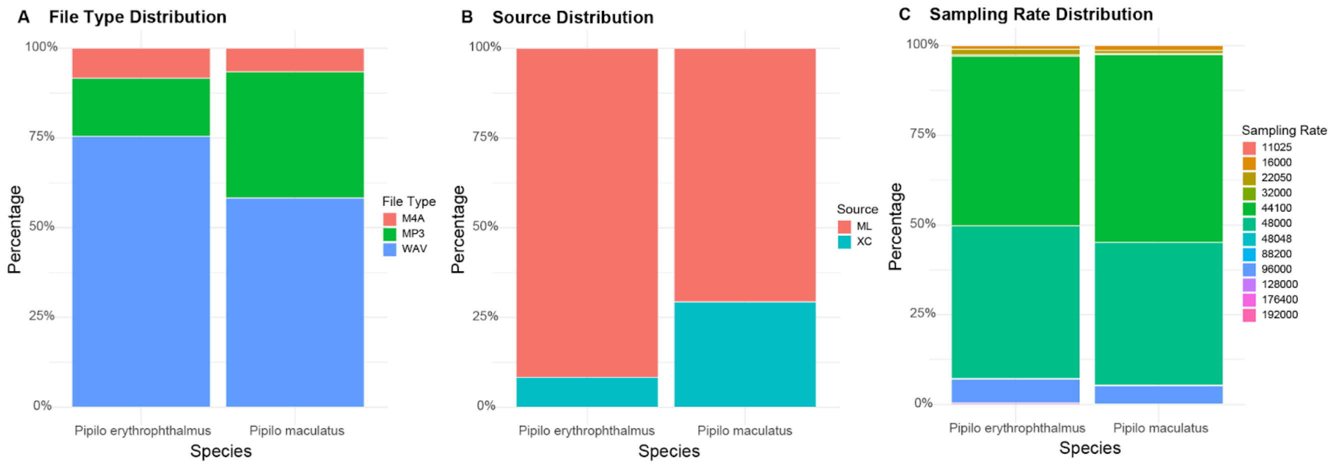

Second, we modified all of our analyses to incorporate conversion status like you suggested, and we find that the changes are very minimal, with a maximum change in species-prediction accuracy of only 1.2%. While conversion status was found to be significant for five song features in our GLM, it does not seem to be useful in making species predictions using any of our tested methods. Below we have included the results of each of these analyses, as well as in the Supplemental Methods and figures S9–S11.

PCA: We conducted a principal component analysis (PCA) in which conversion status was used as a feature alongside the original 16 song features, and we found that conversion status was the second least important feature in PC1 and the least important feature in PC2 . When we fit a linear discriminant analysis (LDA), our species-prediction accuracy remained the same (accuracy = 76.2%; balanced accuracy = 74.0%; kappa = 0.488; $p < 10^{-3}$). Additionally, when color-coding the PC plot by conversion status, there is significant overlap with no apparent separation between whether recordings were converted or not (**see figure below**). We also applied a generalized linear model (GLM) on principal components 1 and 2 with conversion status, scaled longitude, scaled latitude, and species as fixed effects and found that conversion status was *not* statistically significantly associated with PC1 (t-value = 1.11; $p = 0.265$) or PC2 (t-value = 1.10; $p = 0.271$), but longitude and species were significantly associated with both PC1 and PC2 and latitude was significantly associated with PC2.

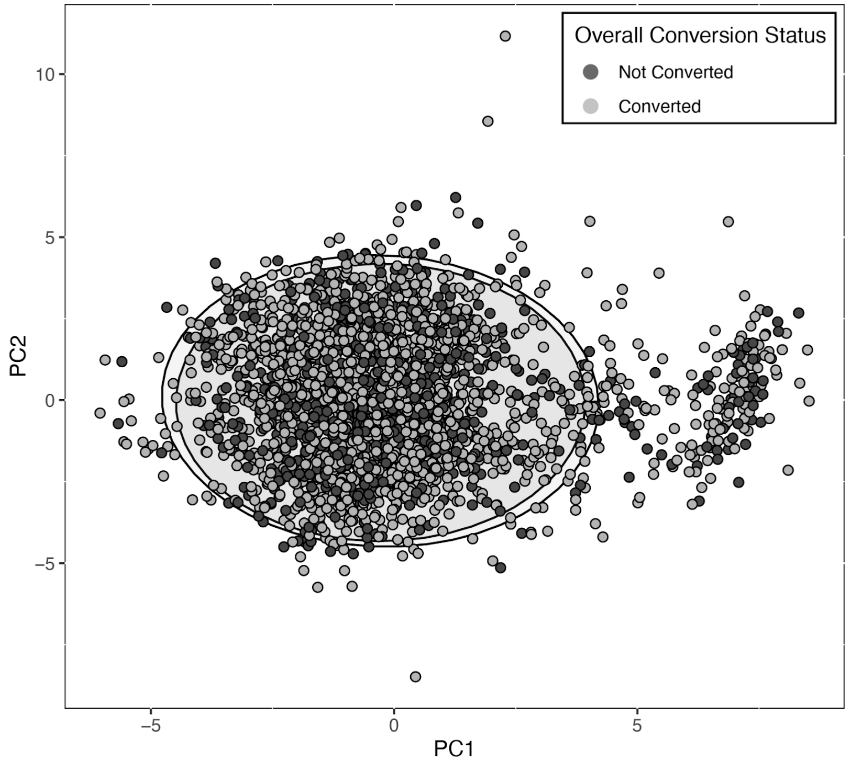

UMAP: Similarly, we then repeated UMAP analysis, this time including conversion status as a feature in the UMAP. When we used LDA to partition the UMAP space to best distinguish Spotted and Eastern towhees, we found that the accuracy changed only by 0.012 (accuracy = 85.2%; balanced accuracy = 84.4%; kappa = 0.687; $p < 10^{-3}$). As with the first two principal components as described above, we applied a GLM to the two dimensions of the UMAP projection and found that conversion status, again, was *not* statistically significant for both UMAP1 (t-value = -0.269; p = 0.788) and UMAP2 (t-value = -1.90; p = 0.058), but species and longitude were significantly associated with UMAP1 and UMAP2, respectively. When color-coding the UMAP plot by conversion status, there is significant overlap with no apparent separation between whether recordings were converted or not (**see figure below**).

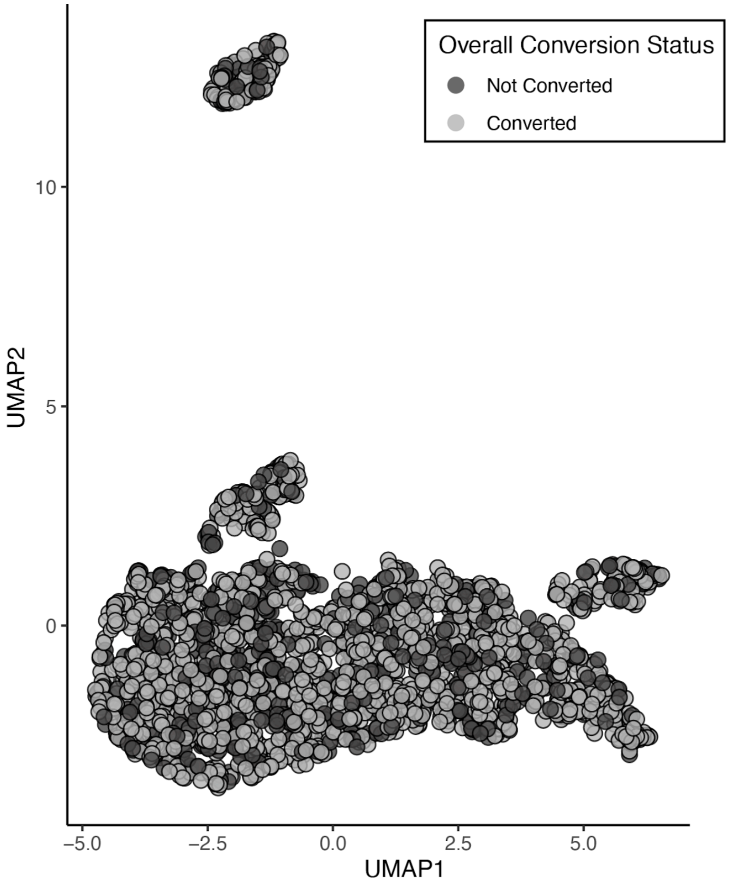

LDA on raw song features: In the LDA using raw song feature data, we incorporated conversion status as an additional feature, and we found that the species-prediction accuracy stayed the same (accuracy = 86.8%; balanced accuracy = 86.8%; kappa = 0.726; *p* = 0.0476). Again, we found that while there was some visible separation of species classification in one-dimensional space, there was still considerable overlap between the 2 species. When color-coding the LD1 plot by conversion status, there is significant overlap with no apparent separation between whether recordings were converted or not (**see figure below**).

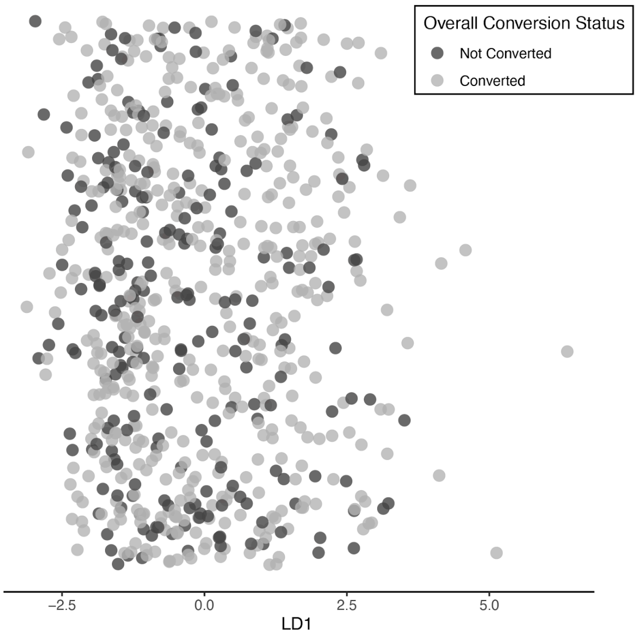

GBM: Additionally, we implemented conversion status as a feature in the gradient boosting machine and found that this feature had the lowest importance, changing the species-prediction accuracy of our model by only 0.001 (accuracy = 88.1%; balanced_accuracy = 88.6%; kappa = 0.755; $p < 10^{-3}$).

RFM 1: We integrated conversion status as a feature in our random forest model 1. We found that overall conversion status was the feature of lowest importance. This model had a 0.3% decrease in accuracy compared to the model in the main text (accuracy = 89.2%; balanced_accuracy = 89.7%; kappa = 0.778; permutation test $p < 10^{-3}$).

RFM 2: We repeated this with random forest model 2 and found that overall conversion status was the feature of lowest importance. When making predictions on the non-overlap zone, the accuracy remained the same (accuracy = 93.5%; balanced_accuracy = 93.6%; kappa = 0.865; permutation test $p < 10^{-3}$). When making predictions on samples from the zone of overlap, the accuracy decreased by 1% (accuracy = 83.3%; balanced_accuracy = 82.6%; kappa = 0.660; permutation test p $< 10^{-3}$), again suggesting it is not an important feature in making predictions.

Specific comments
Fig 1: You normally put more explanation in the figure itself. Having to have to read the figure text to get the axis labels and units is not good enough. This is just screenshots, please include appropriate labeling of axis.

Response: We have updated the figure, which now includes clear axis labels.

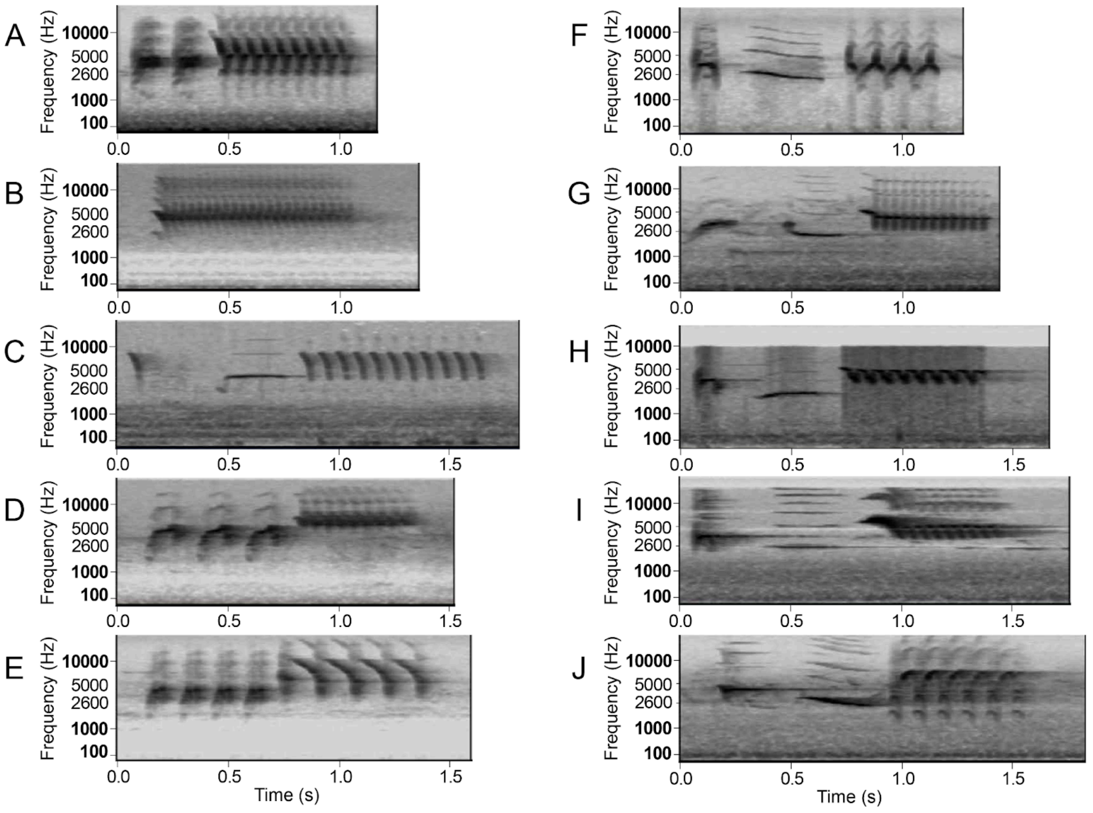

Fig 1. Example spectrograms of Spotted and Eastern towhee songs.
(A-E) Examples of Spotted towhee songs. (F-J) Examples of Eastern towhee songs. The repeated syllable at the end of each song is often called the ‘trill’. The top gray scale shows time in seconds on the x-axis. The scale to the left of the spectrogram shows frequency in Hertz on the y-axis. The number above each spectrogram is the Macaulay Library or Xeno-canto ID number; see metadata (https://github.com/CreanzaLab/TowheeAnalysis) for detailed recording information.

L187 The conversion issue again. When converting from one format to another you can minimize issues by ensuring basic procedures are done, like ensuring a low pass filter of half the new sampling frequency is used. You then avoid aliasing. Please state what you have done to ensure best conversion.

We used Audacity to resample the recordings to 44100 Hz, which keeps the duration of the recording the same and implements a low pass filter at half of the lower sampling rate to avoid aliasing.

New Text:

“Files from Xeno-canto are generally stored in mp3 format and files from Macaulay Library in wav format; we resampled recordings at 44100 Hz if they were recorded at a

different sampling rate and exported each bout as a .wav file to standardize the recordings for analysis, and we noted whether the sampling rate and/or file format had been converted. During resampling, Audacity implements a low-pass filter at half of the sampling rate to prevent aliasing.” L187

L196 This formulation for choice of range is still vague like I commented in the first round.

Response: We have added additional text to further clarify how we define the zone of song overlap. We also would like to note that our goal in this section was to outline the region of “song overlap”—the degrees longitude where both species of towhee have been recorded singing and thus the region where they would be most likely to be exposed to both conspecific and heterospecific song—as opposed to other formulations, such as geographic range overlap defined by sightings of both species or a hybrid zone defined by records of hybridization.

New Text: “To assign the zone of song overlap between the ranges of the two species, for each degree longitude we plotted the proportions of Eastern and Spotted towhees relative to the total number of both species combined, which allowed us to identify where the two species co-occur most frequently and how their relative abundances shift geographically. In other words, at each degree longitude we calculated the number of recordings of Spotted towhees divided by the total number of breeding-season recordings of both species combined, and we repeated this calculation at each degree longitude for the Eastern towhees. We then plotted these proportions on a graph where the x-axis represents longitude and the y-axis represents the proportion of each species (Fig S1). At the most eastern and western longitudes, the recordings were all from Eastern and Spotted towhees, respectively. Based on our findings, we chose to identify the area between 102°W and 91°W as the zone of song overlap, which contained the majority of the area where recordings of both species seemed to co-occur (Fig S1). In addition, we downloaded from eBird all sightings of putative hybrids (eBird query: “Spotted x Eastern Towhee (hybrid) - Pipilo maculatus x erythrophthalmus”) during the breeding season and plotted their locations; we observed that reported hybridization events primarily occurred in this region (Fig S2).” L196

L217 “we adjusted the signal-to-noise threshold, minimum syllable duration, and minimum silence duration to most accurately define the syllables in the song” How did you do that? By some parameter or more subjectively?

Response: In the text, we have clarified that this portion is done subjectively to account for the sometimes dramatic differences in background noise profiles between natural recordings and to safeguard against the possibility that multiple birds are singing at the same time. In our original methods paper about the song analysis techniques used here, we demonstrated that this procedure has high within-user repeatability and high between-user reproducibility (Searfoss et al. 2020).

New Text: “To begin analyzing the features of the syllables within these bouts, we used the Chipper software [37], which was designed to facilitate syllable segmentation and analysis of field recordings with different levels of background noise. This software performs automated syllable segmentation by attempting to parse each song into syllables, defined as periods of sound separated by periods of silence, and then allowing the user to adjust the signal-to-noise ratio, enable high-pass and low-pass filters, and modify the segmentation when syllables have been incorrectly parsed, such as when background noise occurs during the inter-syllable silences (Fig 2). To prevent lower-amplitude syllables from being segmented incorrectly, we normalized the amplitude across each song. Additionally, we adjusted the signal-to-noise threshold, minimum syllable duration, and minimum silence duration to most accurately define the syllables in the song. Since our recordings are from nature and have wide variation in their background noise profiles, we view each spectrogram to assess what is background noise versus the bird singing and manually adjust these parameters so that the syllables are parsed in a way that appears to be most accurately defined with minimal noise; this procedure has been shown to have high within-user repeatability and high between-user reproducibility [37]. If any syllables appear to be parsed incorrectly after this procedure, Chipper allows the user to manually modify the syllable segmentation. If, when visualized in Chipper, we determined that the song overlapped with other birds singing or had excessive background noise, we removed the recording from the analysis. We provide the analyzed song bouts and analysis parameters used in Chipper to extract song features, our code for statistical analysis and visualization of the song feature data, and metadata about each song recording at https://github.com/CreanzaLab/TowheeAnalysis.” L216

Fig 2 Please make the time visualization and text bigger. It is extremely hard to read what it says.

Response: We have updated the time visualization to have larger text.

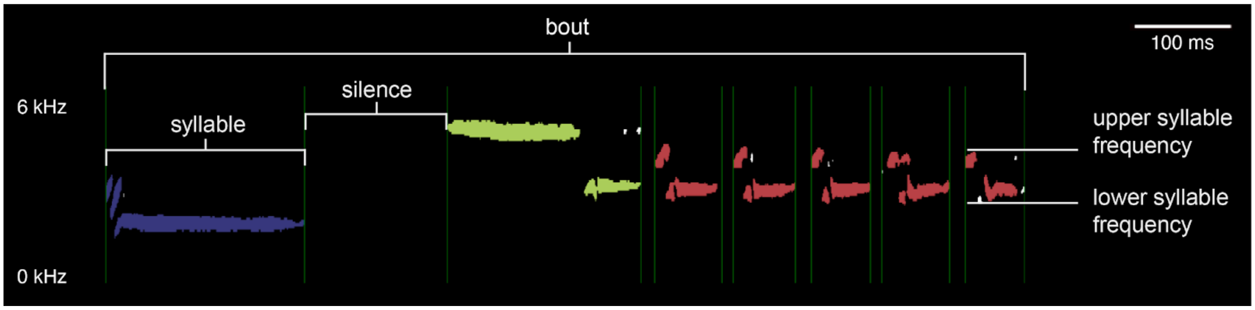

Fig 2. Spectrogram illustrating the definition of specific song elements. On this spectrogram of an Eastern towhee song, we indicate a syllable, silence, bout, maximum syllable frequency, and minimum syllable frequency. The color of the syllable indicates its unique syllable type. Spectrogram generated from Macaulay Library recording 52506381, https://macaulaylibrary.org/asset/52506381.

L291 That equation does not look right in this PDF

Response: On our end, we do not appear to have any issues with the equation in the PDF that we submitted. We have attempted to make sure the equation looks the same on our computer screens compared to others, and we have not encountered any issues. Here is the equation for reference:

$$\frac{1}{2}\left(\frac{number\ of\ correct\ Spotted\ towhee\ classifications}{total\ number\ of\ Spotted\ towhee\ songs\ tested}+\frac{number\ of\ correct\ Eastern\ towhee\ classifications}{total\ number\ of\ Eastern\ towhee\ songs\ tested}\right)$$

L361 This section there is a lot of “We then used command X” without any explanation of what that command does and why you did it.

Response: We have updated the text to explain our methods more clearly and clarify why we are using certain parameters.

Example New Text (updated similarly for each machine learning technique):
“Additionally, we used a deep learning model via the Torch package [45] in R that uses a multilayer perceptron (i.e. fully connected feedforward neural network) for classification of the song bouts. Again, we used a training set consisting of a random balanced subsample of 1592 song bouts ($N_{Spotted\_towhee}$=796 and $N_{Eastern\_towhee}$=796) and tested the model on 697 bouts. The data was centered and scaled prior to integration with the model. The network contains an input layer, two hidden layers, and an output layer. The input layer has a size of 16, defined by the number of song features. We

used 64 neurons in the hidden layers, aiming to balance model performance and computational efficiency without overfitting. Each fully connected layer applies a linear transformation to the data, mapping it to a new space using the ‘nn_linear’ function. The first hidden layer maps the input features to the 64 hidden neurons, followed by a second hidden layer of the same size, both of which use the ReLU activation function to introduce non-linearity. The final fully connected layer outputs the model’s raw predictions, which are then passed through a log-softmax activation function to compute class probabilities. The entire training dataset was passed through the model 100 times (num_epochs = 100) during the training process to allow the model to progressively learn from the training data to improve its accuracy and performance. Within each epoch, we used a small batch size of 32 for more stable convergence, such that 32 training samples were used per iteration so that the model frequently updated its parameters during the training process for more efficient training. We used the ‘nnf_cross_entropy’ loss function for the model to compute the unweighted cross entropy loss for the classification tasks and used an Adam optimization algorithm for Adaptive Moment Estimation with a 0.001 learning rate using the ‘optim_adam’ function to adjust the model’s feature weights during training.” L384

L363 When I suggested Deep Learning I was thinking that you could do it either on the sound, or on the spectrograms, not the features you already extracted. That is the strength of DL networks: To find the relevant parameters by itself. This way you can look at the data from a completely different angle.

Response: Apologies for misunderstanding your previous suggestion! We have since applied a convolutional neural network (CNN) on spectrograms of the towhee recordings. We modified the Audio Classification CNN model from https://github.com/jeffprosise/Deep-Learning. We trained the CNN on spectrogram images from the same balanced set of towhee recordings ($N_{Spotted\_towhee}$=796 and $N_{Eastern\_towhee}$=796) and tested the model on a subset of 697 song bouts that were not in the training set.

We also ran this 10 times using 1) the same 3x3 pixel filters, 2) 5x5 pixel filters, and 3) 7x7 pixel filters, and we calculated the average accuracy of the last epoch for each model, obtaining an average species classification accuracy of 88.32% ± 1.02%, 90.06% ± 1.52%, and 89.74 % ± 1.07%, respectively (Fig. S8).

Our classification accuracies are similar to the other machine learning algorithms we used in this manuscript, which is interesting because the input data are so different (spectrograms vs. parsed song features). Thank you again for this suggestion.

New Text:

Methods: “In addition to the other machine learning models using song feature data, we also applied a convolutional neural network (CNN) on spectrogram images of the towhee recordings to test species classification using a different type of input data and computational approach. We modified an Audio Classification CNN model from [46]. To apply the model to our data, we first created spectrograms from .wav files using the librosa and matplot packages in python and applied low and high pass filters (high pass filter = 1000 Hz; low pass filter = 10000 Hz). Our model accepts images of 224x224 pixels with 3 color channels (RGB) in the input layer. The model then has a series of convolutional layers with 32, 128, 128, and 128 filters, each of size 3x3 pixels, which help to extract increasingly complex features from the input images. Each of the convolutional layers use ReLU activation functions to introduce non-linearity after each convolution. To reduce the dimensions of the feature maps, we use MaxPooling2D layers with a pool size of 2x2 after each convolutional layer to reduce the spatial dimensions of the feature maps, improving efficiency of the model and helping prevent overfitting. The features are then flattened into a one-dimensional vector using the Flatten layer. This vector is then passed to a Dense layer with 1024 neurons and a ReLU activation for further abstraction and learning. The final output layer has 2 neurons (i.e. the Spotted and Eastern towhee classes), using the Softmax activation function to output class probabilities.

To ensure that the results would be comparable to our other models, we used the same training set, such that we trained the CNN on the same balanced set of towhee recordings ($N_{Spotted\_towhee}$=796 and $N_{Eastern\_towhee}$=796). We normalized our data by dividing the input data by 255 pixels and applied one-hot encoding on the labels. We trained the model using the ‘model.fit’ function in Python using a batch size of 32 to update the model’s weights after each set of 32 samples, and we passed the entire training dataset through the network 10 times (epochs = 10). We also repeated this model in three different ways: 1) repeating this exact model 10 times, 2) using a filter size of 5x5 pixels and running that model 10 times, and 3) using a filter size of 7x7 pixels and running that model 10 times to capture a larger section of the images (Fig S8).” L407

Results: “Using a convolutional neural network on spectrogram images of the towhee recordings as an alternative approach to assessing species classification, we obtained an accuracy of 87.52% (balanced accuracy = 88.51%; κ = 0.7457; Permutation test $p < 10^{-3}$), which is similar to the other machine learning algorithms we used in this manuscript (Table 2). We also ran this CNN model 1) 10 times using the same 3x3 filter, 2) 10 times using a 5x5 filter size, and 3) 10 times using a 7x7 filter size, obtaining an average accuracy of 88.32% ± 1.02%, 90.06% ± 1.52%, and 89.74 % ± 1.07%, respectively (Fig S8).” L672

Discussion: “Additionally, our convolutional neural network trained on spectrogram images of the Spotted and Eastern towhee song recordings had similar prediction probabilities as the other machine learning algorithms, suggesting that even when using a different type of classification approach (i.e. raw pixel data versus extracted song features), we obtain similar results.” L777

L364 You start stating that you used an artificial neural network with several hidden layers. That is a good example of what I mean with vague text. Write what network you used and how many layers there is.

Response: We have clarified the neural architecture of our model, including details of each layer and the functions that were used.

New Text: “Additionally, we used a deep learning model via the Torch package [45] in R that uses a multilayer perceptron (i.e. fully connected feedforward neural network) for classification of the song bouts. Again, we used a training set consisting of a random balanced subsample of 1592 song bouts ($N_{Spotted\_towhee}$=796 and $N_{Eastern\_towhee}$=796) and tested the model on 697 bouts. The data was centered and scaled prior to integration with the model. The network contains an input layer, two hidden layers, and an output layer. The input layer has a size of 16, defined by the number of song features. We used 64 neurons in the hidden layers, aiming to balance model performance and computational efficiency without overfitting. Each fully connected layer applies a linear transformation to the data, mapping it to a new space using the ‘nn_linear’ function. The first hidden layer maps the input features to the 64 hidden neurons, followed by a second hidden layer of the same size, both of which use the ReLU activation function to introduce non-linearity. The final fully connected layer outputs the model’s raw predictions, which are then passed through a log-softmax activation function to compute class probabilities. The entire training dataset was passed through the model 100 times (num_epochs = 100) during the training process to allow the model to progressively learn from the training data to improve its accuracy and performance. Within each epoch, we used a small batch size of 32 for more stable convergence, such that 32 training samples were used per iteration so that the model frequently updated its parameters during the training process for more efficient training. We used the ‘nnf_cross_entropy’ loss function for the model to compute the unweighted cross entropy loss for the classification tasks and used an Adam optimization algorithm for Adaptive Moment Estimation with a 0.001 learning rate using the ‘optim_adam’ function to adjust the model’s feature weights during training.” L384

L369 You claim to use a network with 64 layers as that is a multiple of the 16 features. To my knowledge there is no reason to use layers that is a multiple of the number of

features you are investigating. Instead, you add layers if the data becomes more complicated.

Response: Thank you for catching this! We intended to specify that we use 64 neurons in each of our two hidden layers. We have updated the text to clarify this.

New Text: "The first hidden layer maps the input features to the 64 hidden neurons, followed by a second hidden layer of the same size, both of which use the ReLU activation function to introduce non-linearity." L393

L600 In the abstract you say the accuracy of Deep Learning is 89 percent, but here you say 90 percent.

Response: Thank you for catching this! We have updated the abstract to reflect the updated results based on our analyses addressing previous reviewer comments.

Reviewer #3: The authors have done a fantastic job of addressing my original comments, adding two new machine learning analyses, and improving the strength of the other analyses. I only have one more minor comment below.

Response: We appreciate your helpful feedback! We have attempted to address your comment below.

The effects of file conversion have higher p-values (so less significant than other factors) but they also have fairly similar t-values. This indicates that the amplitude of the effect of conversion is at least in the same order of magnitude as other factors, despite being "less significant". I do not think this is a big deal in terms of the interpretations of the results, but the authors should mention it. It may also be briefly worth discussing these effects, as they are relevant to other bioacousticians (I myself am considering mixing WAV and MP3 files in an analysis, and seeing the results of this paper would inform that decision).

Response: We have mentioned this in the main text. We also checked that frequency and timing variables of the individual songs did not change after converting the format or sampling rate (see figure on page 4 above for a visualization). We also ran all the machine learning models again, this time incorporating overall conversion status as features in the models (see Supplemental Methods). We found that the changes are very minimal with a maximum change in accuracy of only 1.2%. Additionally, it was also the feature of lowest importance or second lowest importance in the PCA, GBM, and both RFMs. When color-coding our LDA, PCA, and UMAP plots by overall conversion status, we saw significant overlap with no apparent separation between groups.

Together, this suggests that the overall conversion status does not seem to be useful in making predictions using our machine learning models.

New Text:

Results: “Finally, we assessed whether converting the sampling rate or converting the file type to wav format for consistency had a meaningful effect on the song feature outcomes by including conversion status in the generalized linear models. The models provide evidence that the overall file conversion status was statistically significant only in five song features. For these five features, file conversion had similar t-values to other significant factors, suggesting that the effect of conversion on the song feature has a similar order of magnitude as other factors relative to their standard errors. However, the degree of significance was much less than that of the other variables (i.e. latitude, longitude, and species), indicating that it likely has less predictive power (Table 1).” L505

Discussion: “Since we used two different public repositories of bird song recordings, files had various sampling rates and formats (wav, mp3, and m4a). Because of the potential effect of file type and sampling rate conversion on our analysis, we assessed whether overall conversion status would show statistical significance in our GLM. While the file conversion status had a similar magnitude of effect on the song features as other factors, the degree of significance was much less than the other significant factors in the GLM (Table 1). To investigate the factors underlying this effect, we first verified that the frequency and timing of individual songs did not change after we converted the format or sampling rate; then, we assessed whether the converted files were evenly distributed across our sample, and we found that our database had a higher proportion of Eastern towhee songs in wav format and Spotted towhee songs in mp3 format (Fig S12). Since the song features we studied show significant geographic and species differences, the influence of conversion status in our GLM appears to be related to this biased sampling and not to an effect of conversion on the song features. Further, in addition to the machine learning models reported here, we also ran each model again incorporating overall conversion status as a feature. We found that the changes are very minimal, with a maximum change in accuracy of only 1.2% (see Supplemental Methods). Conversion status was also the feature of lowest importance or second lowest importance in the PCA, GBM, and both RFMs. When color-coding our LDA, PCA, and UMAP plots by overall conversion status, we saw significant overlap with no apparent separation between groups (Figs S9-S11). Together, this suggests that the overall conversion status does not seem to be useful in making species predictions using our machine learning models. ” L857
